# Itaconate confers tolerance to late NLRP3 inflammasome activation

**DOI:** 10.1016/j.celrep.2021.108756

**Published:** 2021-03-09

**Authors:** Monika Bambouskova, Lucie Potuckova, Tomas Paulenda, Martina Kerndl, Denis A. Mogilenko, Kate Lizotte, Amanda Swain, Sebastian Hayes, Ryan D. Sheldon, Hyeryun Kim, Unnati Kapadnis, Abigail E. Ellis, Christine Isaguirre, Samantha Burdess, Anwesha Laha, Gaya K. Amarasinghe, Victor Chubukov, Thomas P. Roddy, Michael S. Diamond, Russell G. Jones, Donald M. Simons, Maxim N. Artyomov

**Affiliations:** 1 Department of Pathology and Immunology, Washington University School of Medicine, St. Louis, MO 63110, USA; 2 Department of Medicine, Washington University School of Medicine, St. Louis, MO 63110, USA; 3 Institute for Vascular Biology, Centre for Physiology and Pharmacology, Medical University Vienna, 1090 Vienna, Austria; 4 Christian Doppler Laboratory for Arginine Metabolism in Rheumatoid Arthritis and Multiple Sclerosis, 1090 Vienna, Austria; 5 Agios Pharmaceuticals, 88 Sidney Street, Cambridge, MA 02139, USA; 6 Van Andel Research Institute, Metabolic and Nutritional Programming, Center for Cancer and Cell Biology, Grand Rapids, MI 49503, USA; 7 Department of Molecular Microbiology, Washington University School of Medicine, St. Louis, MO 63110, USA; 8 The Andrew M. and Jane M. Bursky Center for Human Immunology and Immunotherapy Programs, Washington University School of Medicine, St. Louis, MO 63110, USA; 9 Lead contact

## Abstract

Itaconate is a unique regulatory metabolite that is induced upon Toll-like receptor (TLR) stimulation in myeloid cells. Here, we demonstrate major inflammatory tolerance and cell death phenotypes associated with itaconate production in activated macrophages. We show that endogenous itaconate is a key regulator of the signal 2 of NLR family pyrin domain containing 3 (NLRP3) inflammasome activation after long lipopolysaccharide (LPS) priming, which establishes tolerance to late NLRP3 inflammasome activation. We show that itaconate acts synergistically with inducible nitric oxide synthase (iNOS) and that the ability of various TLR ligands to establish NLRP3 inflammasome tolerance depends on the pattern of co-expression of IRG1 and iNOS. Mechanistically, itaconate accumulation upon prolonged inflammatory stimulation prevents full caspase-1 activation and processing of gasdermin D, which we demonstrate to be post-translationally modified by endogenous itaconate. Altogether, our data demonstrate that metabolic rewiring in inflammatory macrophages establishes tolerance to NLRP3 inflammasome activation that, if uncontrolled, can result in pyroptotic cell death and tissue damage.

## In brief

Bambouskova et al. determine the *in vitro* phenotype of *Irg1^−/−^* macrophages and define itaconate as a key regulator of tolerance to late NLRP3 inflammasome activation.

## Graphical Abstract

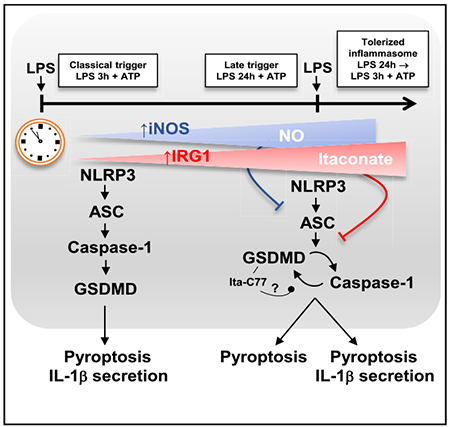

## INTRODUCTION

Aconitate decarboxylase 1 (encoded by *Acod1* or Immune-responsive gene 1; *Irg1*) is the enzyme solely responsible for itaconate production by activated macrophages (Michelucci et al., 2013). Upon lipopolysaccharide (LPS) activation, induction of *Irg1* expression leads to accumulation of substantial (millimolar) amounts of itaconate (Jha et al., 2015; Michelucci et al., 2013), a metabolite that plays an important role in remodeling the tricarboxylic acid (TCA) cycle and inhibits succinate dehydrogenase (SDH) (Cordes et al., 2016; Lampropoulou et al., 2016). In addition, endogenous itaconate produced by activated macrophages has been shown to activate NRF2- and ATF3-driven responses (Bambouskova et al., 2018). Immunologically, we previously reported that itaconate mildly suppresses NLR family pyrin domain containing 3 (NLRP3) inflammasome-mediated interleukin (IL)-1β secretion using *Irg1^−/−^* macrophages (Lampropoulou et al., 2016). More recently, we demonstrated that pre-treatment of the macrophages with non-derivatized itaconate leads to inhibition of the NLRP3 inflammasome at the level of signal 2 (Swain et al., 2020). However, the details of IL-1β secretion regulation by non-derivatized/endogenous itaconate are not understood. Although itaconate reaches high intracellular concentrations in activated macrophages, its regulatory effect on IL-1β secretion appears to be relatively modest based on studies with itaconate-deficient *Irg1^−/−^* macrophages after LPS treatment (Lampropoulou et al., 2016; Swain et al., 2020). This raises the more general question of why macrophages undergo such a substantial metabolic commitment to itaconate production, even though its regulatory impact appears to be disproportionately small. One consideration that reconciles these observations is the possibility that LPS activation or conventional NLRP3 inflammasome activation by itself might not be the optimal context to reveal the key functional aspects of itaconate’s immunoregulatory potential.

Here, we introduce an alternative experimental design and report marked differences between wild-type (WT) and *Irg1^−/−^* macrophages *in vitro*. Specifically, we find that itaconate regulates LPS-induced tolerance to late pyroptosis and late NLRP3 inflammasome activation. Using this experimental design, we link itaconate and inducible nitric oxide synthase (iNOS) immunomodulatory activities, highlight the critical regulatory role of gasdermin D (GSDMD) in late NLRP3 inflammasome activation, and reveal direct post-translational modification (PTM) of GSDMD by endogenous itaconate.

## RESULTS

### *Irg1* establishes LPS-mediated tolerance to signal 2 of NLRP3 inflammasome activation

**Figure 1.**
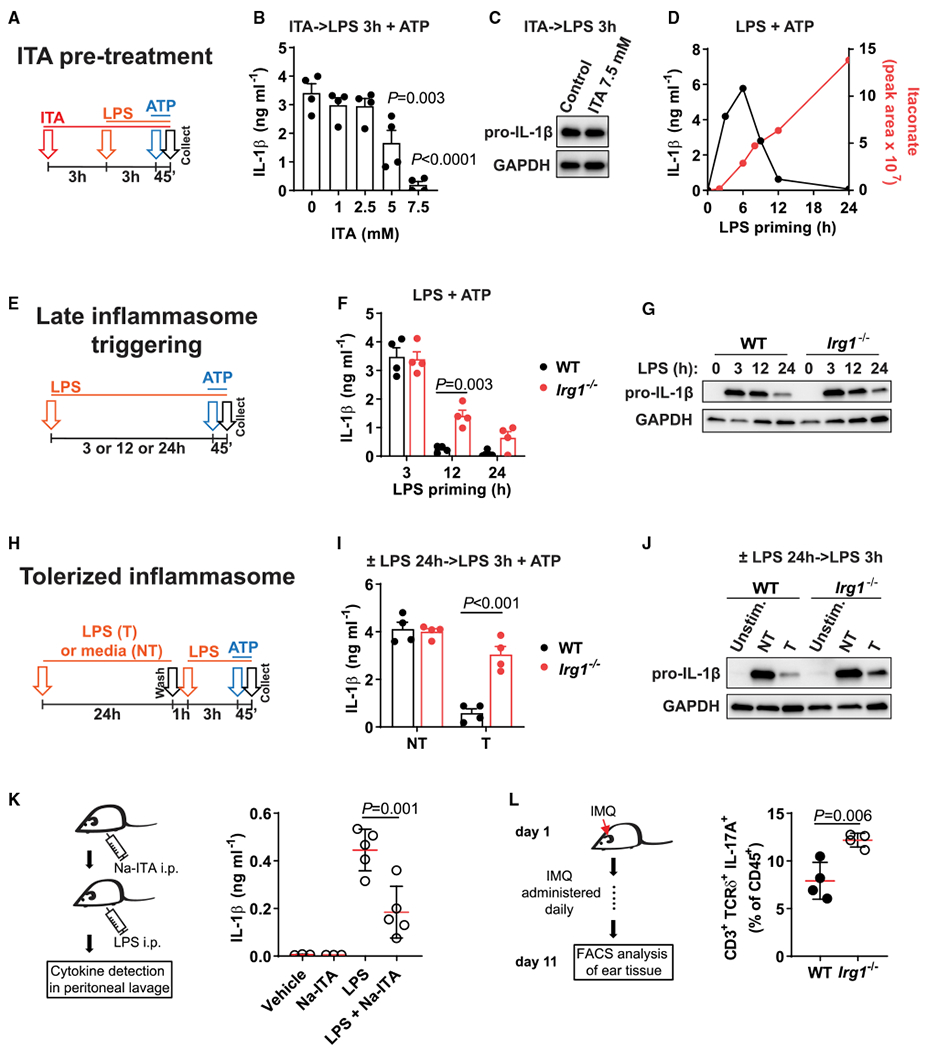
*Irg1* establishes LPS-mediated tolerance to signal 2 of NLRP3 inflammasome activation (A) Design of BMDM pre-treatment with itaconate (ITA, 3 h) and classical NLRP3 inflammasome triggering: LPS priming for 3 h followed by ATP (45 min). (B and C) IL-1β protein detection in supernatants (B) or cell lysates (C) of BMDMs treated as in (A). No ATP was added in (C). (D) Overlay of the time course of IL-1β secretion in BMDMs primed with LPS for the indicated time followed by ATP, and intracellular ITA levels in BMDMs stimulated with LPS. ITA data from Swain et al. (2020). (E) Design of late inflammasome triggering. Cells are primed with LPS for 3 h (classical triggering) or 12 or 24 h (late triggering) followed by ATP. (F and G) IL-1β protein detection in supernatants (F) or cell lysates (G) of BMDMs treated as in (E). No ATP was added in (G). (H) Design of inflammasome tolerization. (I and J) IL-1β protein detection in supernatants (I) or cell lysates (J) of BMDMs treated as in (H). No ATP was added in (J). (K) Mice were injected with sodium itaconate (Na-ITA, 1.24 mg) i.p. 20 and 4 h before LPS. After LPS challenge (4 mg kg^−1^, 6 h) IL-1β was analyzed in peritoneal fluid. Data are from n = 3 vehicle, Na-ITA mice and n = 5 LPS, Na-ITA + LPS mice. (L) Scheme of cutaneous IMQ administration and analysis of cell frequencies in mouse ear tissue determined by flow cytometry, with n = 4 mice per group. Western blots are representative of n = 3 experiments. GAPDH was used as loading control. T, tolerized; NT, non-tolerized; Unstim., unstimulated control. In (B), (F), and (I), n = 4 experiments. Data represent mean ± SEM. The p values were calculated using (B and K) 1-way ANOVA with Tukey’s test, (F and I) 2-way ANOVA with Sidak’s test, and (L) unpaired, two-tailed Student’s t test. See also Figure S1.

We previously demonstrated that itaconate inhibits NLRP3 inflammasome activation in macrophages (Lampropoulou et al., 2016; Swain et al., 2020). For instance, pre-treatment of bone marrow-derived macrophages (BMDMs) with the native, non-derivatized form of itaconate before classical NLRP3 inflammasome activation (priming with LPS for 3 h, signal 1, followed by treatment with adenosine triphosphate [ATP], signal 2; Swanson et al., 2019) significantly downregulates IL-1β secretion (Swain et al., 2020) (Figure 1A). Consistent with our prior observations, itaconate strongly inhibited IL-1β secretion when added at concentrations of 5–7.5 mM (Figure 1B), whereas the intracellular levels of pro-IL-1β remained unchanged (Figure 1C). Thus, we evaluated the effect of itaconate on key events associated with signal 2, starting with apoptosis-associated speck-like protein containing a caspase recruitment domain (ASC) speck formation. Using ASC-mCitrine-expressing BMDMs (Tzeng et al., 2016), we found that itaconate pre-treatment did not affect ASC speck formation upon NLRP3 inflammasome triggering, whereas a known NLRP3 inhibitor, MCC950 (Coll et al., 2015), blocked ASC speck formation (Figures S1A–S1D). These data indicate that itaconate interferes with signal 2 events downstream of NLRP3 activation.

We next evaluated whether this difference in IL-1β secretion upon pre-treatment with itaconate can be translated into a similarly robust phenotype in *Irg1*-deficient macrophages. As itaconate begins to accumulate in BMDMs, starting from 4 to 6 h after LPS treatment, we reasoned that the conventional NLRP3 inflammasome activation protocol (LPS 3 h + ATP) might not be the optimal assay to understand the effects of endogenous itaconate on IL-1β secretion. Therefore, we examined NLRP3 inflammasome functionality after longer LPS priming. IL-1β secretion peaks 3 to 6 h after LPS priming and then shuts down after 12 h or more, which inversely correlates with levels of accumulating itaconate in the cells (Figure 1D, mass spectrometry data from Swain et al., 2020). Therefore, we compared WT and *Irg1^−/−^* macrophages with different LPS priming durations (classical NLRP3 inflammasome activation with 3 h of LPS priming or late NLRP3 activation after 12 to 24 h of LPS priming) (Figure 1E). Notably, *Irg1^−/−^* cells continued to secrete high levels of IL-1β upon late NLRP3 activation (~12 h) (Figure 1F). At the same time, pro-IL-1β levels were identical between WT and *Irg1^−/−^* cells at 12 h after LPS priming (Figure 1G; Figure S1E). These data indicate that endogenous and exogenous itaconate interferes with NLRP3 inflammasome events associated with signal 2.

The decrease in IL-1β secretion after 24 h of LPS priming correlates with the decrease in pro-IL-1β levels in both *Irg1^−/−^* and WT macrophages (Figure 1G), which is consistent with previous reports of prolonged LPS stimulation before inflammasome activation (Guarda et al., 2011; Hernandez-Cuellar et al., 2012). Because pro-IL-1β levels are determined by events associated with signal 1, we evaluated what happens when macrophages are restimulated with LPS+ATP after 24 h of LPS pre-stimulation (Figure 1H). RNA sequencing (RNA-seq) data confirmed that there is a robust boost in *Il1b* expression upon LPS restimulation (Figure S1H). We found that IL-1β secretion in WT macrophages was downregulated upon secondary challenge with LPS+ATP (Figure 1I). We refer to this phenomenon as endotoxin-induced tolerance to inflammasome activation, reminiscent of endotoxin tolerance to LPS restimulation (Foster et al., 2007; Medzhitov et al., 2012). However, in LPS-tolerized *Irg1^−/−^* macrophages, LPS+ATP restimulation resulted in IL-1β secretion that was comparable to classically activated, non-tolerized macrophages (Figure 1I). The same trend was observed for another inflammasome-dependent cytokine, IL-18, for which the secretion in tolerized *Irg1^−/−^* macrophages reached even higher levels than in non-tolerized macrophages (Figure S1G). Despite the large difference in IL-1β secretion, pro-IL-1β levels and production of inflammasome-independent cytokines were similar between WT and *Irg1^−/−^* macrophages (Figure 1J; Figures S1F, S1H, and S1I), again suggesting that the effect of endogenous itaconate in these settings is associated with signal 2 of inflammasome activation.

Next, we confirmed that the itaconate pre-treatment also downregulates IL-1β secretion in human monocyte-derived macrophages (MoDMs) (Figure S1J). Human MoDMs downregulated IL-1β secretion upon late NLRP3 activation, as observed in mouse WT cells, suggesting that the LPS-mediated tolerance to delayed NLRP3 activation is conserved in human macrophages (Figure S1K). The inhibitory effect of itaconate on inflammasome activation was also evident *in vivo* using two distinct models. First, intraperitoneal (i.p.) administration of sodium itaconate prevented IL-1β secretion in the peritoneum after i.p. LPS injection, whereas IL-6 production was not affected (Figure 1K; Figure S1L). IL-1β in these settings has been shown to be NLRP3 dependent (Coll et al., 2015). Second, we evaluated the effect of IRG1 deficiency in the setting of low-level, persistent inflammation *in vivo* using the imiquimod (IMQ)-induced model of psoriasis. *Irg1^−/−^* mice had higher proportions of IL-17A-producing T cell receptor delta-expressing (TCRδ^+^) T cells in the ear, which are known to depend on IL-1β release by myeloid cells (Coll et al., 2015; McGinley et al., 2020). However, CD45^+^, CD11b^+^ Ly6G^+^ neutrophils; CD11b^+^ Ly6c^+^ monocytes; CD11c^+^ MHCII^+^ dendritic cells; and CD4^+^ T cell infiltration did not increase in *Irg1^−/−^* animals (Figure 1L; Figure S1M). Altogether, these data establish IRG1 as a key regulatory factor mediating endotoxin tolerance to NLRP3 inflammasome activation.

### *Irg1* phenotype is associated with dysregulation of caspase-1 and GSDMD processing

We showed that IRG1 regulates tolerance to inflammasome activation after LPS stimulation; however, it is unclear whether this effect is specific to the NLRP3 inflammasome or whether other inflammasome complexes can also be tolerized by itaconate. Therefore, we tested whether IRG1 expression induces tolerance in the context of different inflammasomes. When absent in melanoma 2 (AIM2) inflammasome was stimulated in non-tolerized or LPS-tolerized cells, we observed the IL-1β secretion was only mildly inhibited in tolerized cells and *Irg1^−/−^* macrophages did not show further increase in IL-1β secretion (Figure S2A), suggesting that the tolerance is specific to NLRP3 inflammasome. Following this line of investigation, we also tested non-canonical NLRP3 stimulation and found IL-1β secretion to be fully tolerized in both WT and *Irg1^−/−^* macrophages (Figure S2B), conferring further specificity of the effect of IRG1 on canonical NLRP3 inflammasome activation.

**Figure 2.**
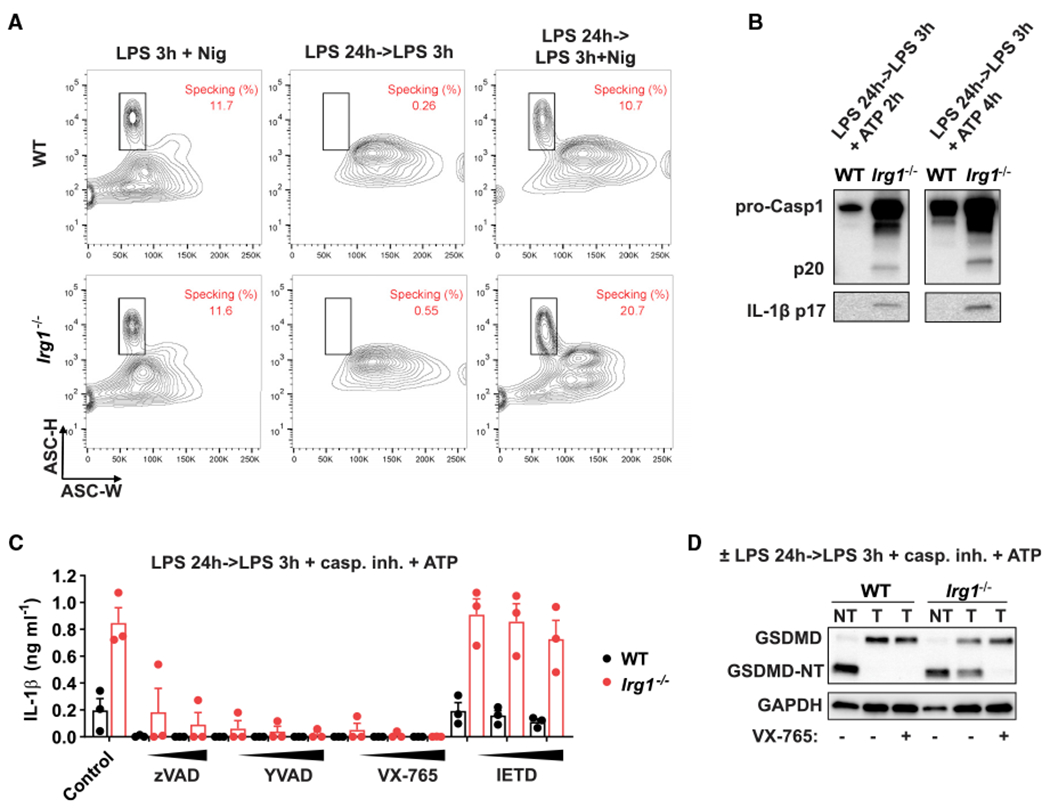
*Irg1* phenotype is associated with dysregulation of caspase-1 and GSDMD processing (A) BMDMs were stimulated as in Figure 1H. Nigericin (Nig) for 1 h was used to trigger inflammasome. Fixed and permeabilized cells were analyzed for ASC scattering properties by flow cytometry. (B) Protein levels analyzed by western blot in supernatants of BMDMs stimulated as in Figure 1H, ATP was added for the indicated time. (C) IL-1β secretion in BMDMs treated as in Figure 1H. Caspase inhibitors (20, 50, and 100 μM) were added 30 min before ATP. Data are mean ± SEM, with n = 3 experiments. (D) GSDMD detection in cell lysates of BMDMs treated as in Figure 1H. VX-765 (caspase-1 inhibitor, 50 μM) was added 30 min before ATP. Western blots are representative of n = 3 experiments. GAPDH was used as loading control. See also Figure S2.

Next, we characterized key signaling events in WT and IRG1-deficient macrophages in the inflammasome tolerance settings with a focus on the canonical NLRP3 inflammasome. First, analysis of ASC specking demonstrated that triggering late inflammasome induces a significant amount of ASC specking even in itaconate-producing WT macrophages (Figure 2A). This indicates that itaconate’s impact is downstream of ASC specking. *Irg1^−/−^* macrophages demonstrated an increased level of ASC specking, consistent with the ability to fully activate inflammasome after LPS tolerization (Figure 2A; Figure S2C). Indeed, tolerized *Irg1^−/−^* macrophages showed upregulated processing of caspase-1 and IL-1β (Figure 2B). To evaluate the dependence of the phenotype on caspase-1, we tested several caspase inhibitors (zVAD = pan-caspase, YVAD = caspase-1, VX-765 = caspase-1, IETD = caspase-8); only caspase-1 inhibitors blocked IL-1β secretion in tolerized *Irg1^−/−^* macrophages (Figure 2C). Next, we found that GSDMD processing into its N-terminal part (GSDMD-NT) is markedly different in *Irg1^−/−^* cells; tolerized WT cells did not exhibit GSDMD processing, whereas *Irg1^−/−^* cells showed effective GSDMD cleavage (Figure 2D), which was also blocked by the addition of the caspase-1 inhibitor. However, the effect was not likely due to direct inhibition of caspase-1 p20 activity by itaconate based on *in vitro* enzyme activity assays with recombinant p20, because modest inhibition could be observed only at the unphysiologically high concentration of 20 mM itaconate (Figure S2D).

Itaconate was previously reported to inhibit SDH activity (Lampropoulou et al., 2016) and trigger the NRF2 and ATF3 transcription factors in activated macrophages (Bambouskova et al., 2018). We tested whether genetic deficiency in any of these pathways could phenocopy the lack of NLRP3 inflammasome tolerance observed in *Irg1^−/−^* macrophages. First, we evaluated the effect of SDH using tamoxifen-inducible SDH knockout (*Sdhb*^fl/fl^) macrophages; however, these cells did not recapitulate the *Irg1^−/−^* phenotype (Figures S2E and S2F). Similarly, neither *Nrf2*- nor *Atf3*-deficient macrophages phenocopied the *Irg1^−/−^* macrophages, because both genotypes demonstrated robust inflammasome tolerance upon LPS restimulation (Figures S2G and S2H), suggesting that IRG1 does not act on the inflammasome solely through these factors. Altogether, these data establish that IRG1 mediates tolerance to NLRP3 inflammasome activation due to so-far-unidentified mechanisms.

### Itaconate reconstitution rescues NLRP3 inflammasome tolerance in *Irg1*-deficient macrophages

**Figure 3.**
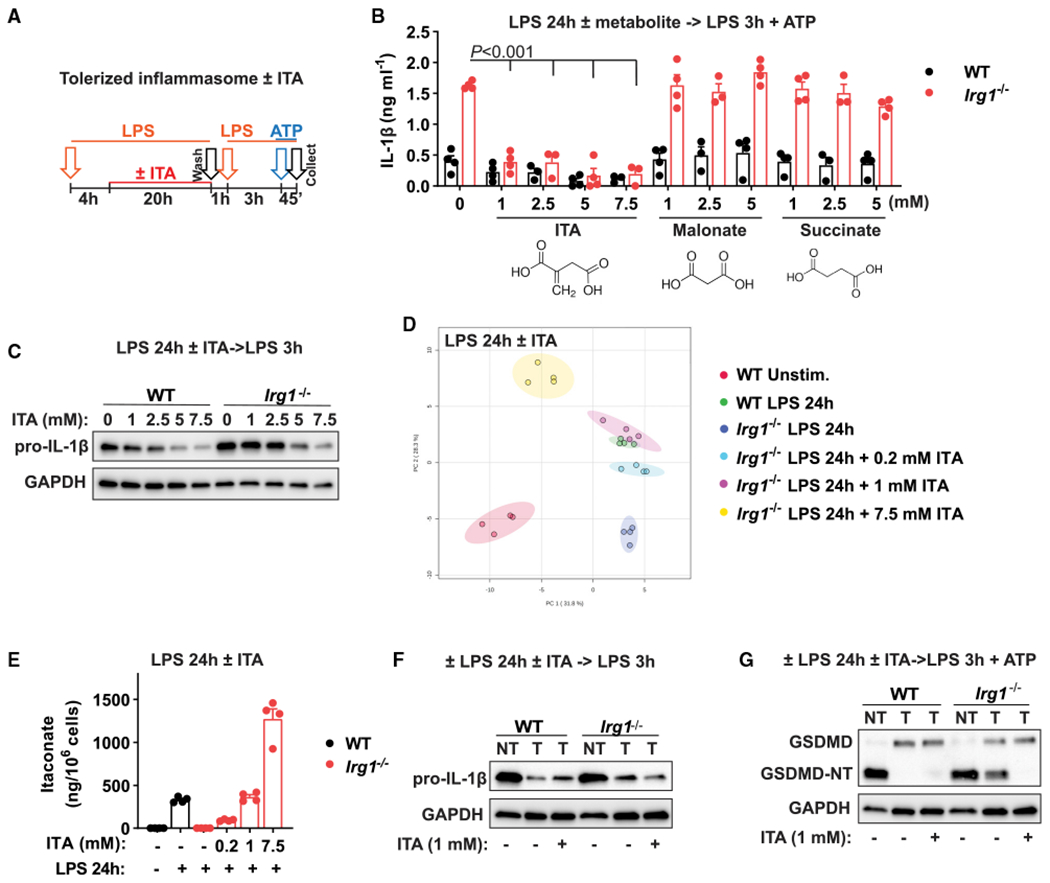
ITA reconstitution rescues NLRP3 inflammasome tolerance in *Irg1*-deficient macrophages (A) Design of inflammasome tolerization and ITA reconstitution (added at 4 h of LPS pre-stimulation). (B) IL-1β secretion in BMDMs stimulated and treated as in (A). ITA or other metabolites were added as indicated, with n = 3–4 experiments. The p values were calculated using 2-way ANOVA with Sidak’s test. (C) Pro-IL-1β detection in cell lysates of BMDMs treated and stimulated as in (A). No ATP was added. (D) PCA of global metabolic profiles of BMDMs stimulated with LPS for 24 h and reconstituted with ITA at 4 h of stimulation, with n = 4 cultures. (E) ITA quantification in samples from (D). (F and G) Protein detection in BMDM lysates treated and stimulated as in (A). No ATP was added in (F). Western blots are representative of n = 3 experiments. GAPDH was used as loading control. Data represent mean ± SEM. See also Figure S3 and Table S1.

To determine whether itaconate production or some other function of the IRG1 enzyme explains the observed NLRP3 inflammasome tolerance phenotype, we reconstituted IRG1-deficient macrophages with itaconate. To avoid possible interference with the early Toll-like receptor (TLR) signaling events and to mimic the inducible character of itaconate production, itaconate was added 4 h after the initial LPS stimulation (Figure 3A). Adding itaconate to *Irg1^−/−^* cells rescued the tolerance phenotype; i.e., IL-1β was not produced after late NLRP3 triggering (Figure 3B). Itaconate reconstitution already inhibited IL-1β secretion when added at 1 mM concentration, whereas adding 1 mM itaconate in the absence of prolonged LPS stimulation (Figure S3A) did not result in effects on IL-1β secretion. This indicates that itaconate’s regulatory potential is significantly enhanced in an inflammatory context (i.e., ongoing LPS signaling). Furthermore, the addition of structurally similar metabolites, malonate and succinate, did not inhibit IL-1β secretion in *Irg1^−/−^* macrophages (Figure 3B). Because malonate is also a known inhibitor of SDH in macrophages (Swain et al., 2020), these results supplement the data from *Sdh*-deficient cells (Figure S2F) and confirm that the observed phenotype is independent of SDH activity. At higher reconstitution concentrations (e.g., 5 to 7.5 mM), itaconate starts to inhibit expression of pro-IL-1β (Figure 3C; Figure S3B), underscoring that in a proinflammatory setting, itaconate’s impact is considerably enhanced compared with the pre-treatment of resting macrophages with itaconate (Figure 1C).

We next determined the concentration of exogenously added itaconate that best mimics endogenous production of itaconate by activated WT macrophages. To that end, we performed metabolic profiling of intracellular itaconate at different concentrations of reconstituted itaconate. Principal-component analysis (PCA) of metabolic data demonstrated global similarity of reconstitution with 1 mM itaconate to the metabolic state of activated WT macrophages (Figure 3D; Figure S3C; Table S1). Furthermore, addition of 1 mM itaconate nearly matched the levels of itaconate in WT cells upon 24 h of LPS stimulation (Figure 3E), indicating that addition of this itaconate concentration results in physiological intracellular levels. Functionally, reconstitution at this concentration affected only IL-1β secretion, not pro-IL-1β levels (Figures 3B and 3F); blocked GSDMD processing in tolerized *Irg1^−/−^* cells; and inhibited caspase-1 activation without affecting total protein levels (Figure 3G; Figures S3D and S3E), thus reflecting the behavior of activated WT macrophages. Furthermore, reconstitution with 1 mM exogenous itaconate in LPS-activated, IRG1-deficient macrophages reproduced effects of itaconate on NRF2 and ATF3 (Figure S3F), which we previously showed to occur in an inflammatory context, but not upon pre-treatment of resting macrophages (Bambouskova et al., 2018; Swain et al., 2020). Altogether, these data demonstrate that itaconate accumulation in activated macrophages causes late inflammasome tolerance through the previously undescribed mechanism.

### Itaconate synergizes with iNOS to tolerize NLRP3 inflammasome activation

**Figure 4.**
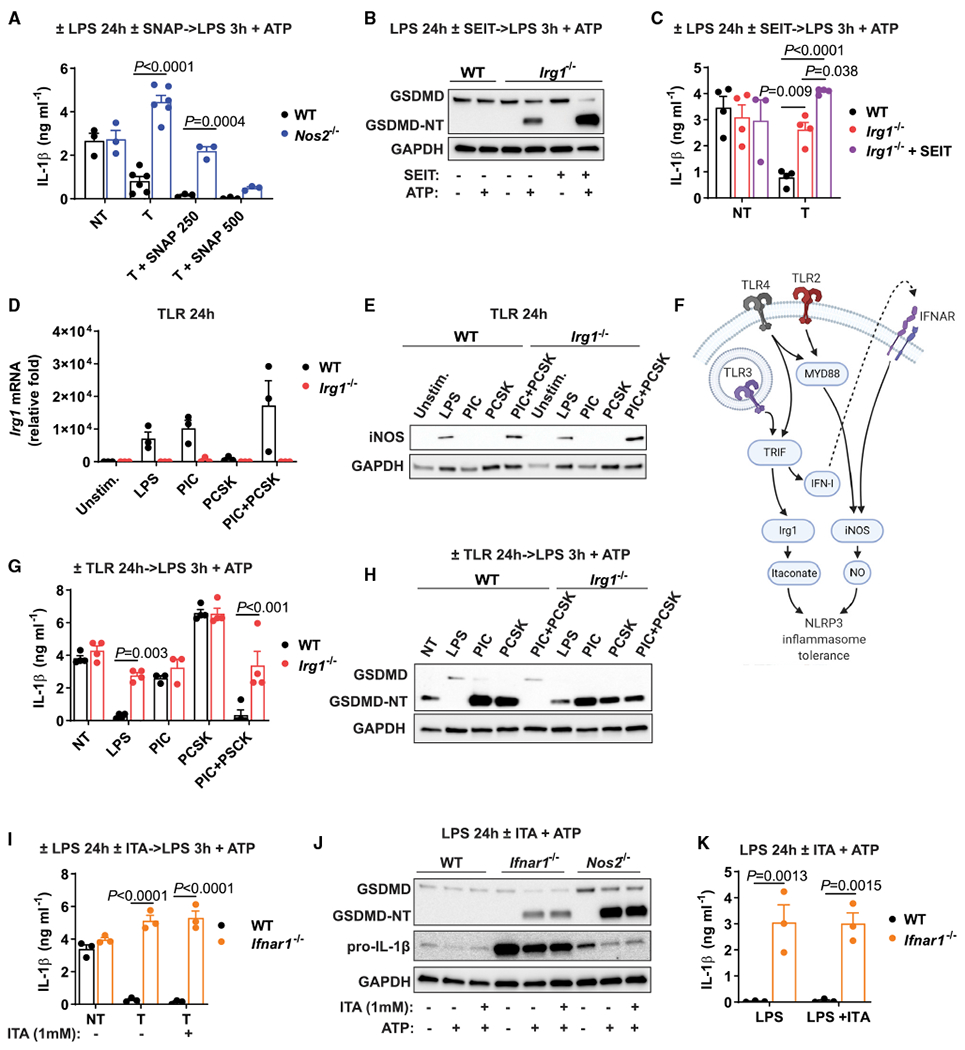
ITA synergizes with iNOS to tolerize NLRP3 inflammasome activation (A) IL-1β secretion in BMDMs stimulated as in Figure 3A. SNAP (in micromolars) was added instead of ITA at 4h of LPS pre-treatment, with n = 3–6 experiments. (B) GSDMD detection in cell lysates of BMDMs treated as in Figure 1H. SEIT was added at 1 h of LPS pre-stimulation. (C) IL-1β secretion in BMDMs stimulated as in (B), with n = 3–4 experiments. (D) *Irg1* mRNA levels in BMDMs stimulated with TLR ligands for 24 h, with n = 3 experiments. (E) iNOS protein detection in lysates of BMDMs stimulated as in (D). (F) Model of signaling pathways controlling expression of inflammasome tolerance. (G) IL-1β secretion in BMDMs stimulated as in Figure 1H. Pre-stimulation with various TLR ligands as indicated, with n = 3–4 experiments. (H) GSDMD detection in cell lysates of BMDMs stimulated as in (G). (I) IL-1β secretion in BMDMs treated and stimulated as in Figure 3A, with n = 3 experiments. (J) Protein detection in lysates of BMDMs stimulated with LPS for 24 h followed by ATP. ITA was added at 4 h of LPS stimulation. (K) IL-1β secretion in BMDMs treated as in (J), with n = 3 experiments. Western blots are representative of n = 3 experiments. GAPDH was used as loading control. Data represent mean ± SEM. The p values were calculated using 2-way ANOVA with Sidak’s test. See also Figure S4.

In *Irg1^−/−^* macrophages, GSDMD is processed only partially (Figure 2D; see also Figure 3G) during late inflammasome activation. Although even this partial cleavage of GSDMD is sufficient to support potent IL-1β secretion, it suggests that additional mechanisms might contribute to late NLRP3 inflammasome tolerance. iNOS has been described as an inhibitor of inflammasomes in later stages of macrophage activation (Hernandez-Cuellar et al., 2012; Mao et al., 2013; Mishra et al., 2013). Indeed, we observed that *Nos2^−/−^* macrophages were not able to establish NLRP3 inflammasome tolerance upon LPS stimulation, and this was reversed by the addition of the nitric oxide (NO) donor S-nitroso-N-acetylpenicillamine (SNAP) (Figure 4A). This result defines iNOS as another key factor establishing NLRP3 inflammasome tolerance in LPS-activated macrophages.

We next explored the requirements for iNOS activity and itaconate production in the context of NLRP3 inflammasome tolerance. *Nos2^−/−^* macrophages upregulate IRG1 to the same extent as activated WT macrophages, and robust production of itaconate in these cells has been shown previously (Bailey et al., 2019). Moreover, activated *Irg1^−/−^* macrophages express high levels of iNOS, matching its upregulation in activated WT macrophages (Figure S4A). Because deficiency in either of these genes disrupts inflammasome tolerance, we hypothesized that the presence of both factors is required for LPS-mediated tolerance to NLRP3 activation. To explore this idea, we tested NLRP3 activation in *Irg1^−/−^* macrophages in which iNOS action was blocked with the pharmacological inhibitor S-ethylisothiourea (SEIT) (Figure S4B). Absence of iNOS activity further increases NLRP3 activation in *Irg1^−/−^* macrophages, as shown by GSDMD processing and IL-1β secretion (Figures 4B and 4C; Figures S4C and S4D). Consistent with the *Nos2^−/−^* data (Figure 4A), in the absence of iNOS activity, itaconate reconstitution was not sufficient to induce inflammasome tolerance (Figures S4E and S4F). Collectively, these data establish iNOS as a key co-regulator of NLRP3 inflammasome tolerance that synergizes with itaconate.

### IRG1-iNOS synergy defines NLRP3 inflammasome tolerance for different TLR stimulations

We next explored the signaling pathways responsible for the tolerance to NLRP3 inflammasome activation. We evaluated patterns of *Irg1* mRNA and iNOS expression upon stimulation with different TLR ligands and compared them with the ability to establish inflammasome tolerance. *Irg1* mRNA was detected upon LPS (TLR4) and poly(I:C) (PIC; TLR3) but not Pam3CSK4 (PCSK; TLR2/TLR1) stimulation, whereas iNOS protein levels were detected upon LPS stimulation only (Figures 4D and 4E). This pattern reflects that *Irg1* induction depends on TRIF stimulation, whereas upregulation of iNOS protein depends on engagement of both TRIF and MyD88 adaptors (Farlik et al., 2010; Hirotani et al., 2005). Accordingly, the combination of PIC and PCSK induced expression of both *Irg1* and iNOS (Figures 4D and 4E). Based on the observed functional synergy between itaconate and NO, we hypothesized that TLR-induced inflammasome tolerance occurs only when both MyD88 and TRIF pathways are engaged (Figure 4F). Only LPS or PIC+PCSK stimulation led to full tolerance of WT macrophages (but not *Irg1^−/−^* macrophages), as evident by higher levels of IL-1β secretion and GSDMD processing (Figures 4G and 4H).

Given that type I interferon (IFN-I) signaling induces expression of iNOS protein (Farlik et al., 2010; Hirotani et al., 2005) (Figure 4F), we next tested its role in establishing NLRP3 inflammasome tolerance. For this purpose, we interrogated *Ifnar1^−/−^* macrophages, which induce normal levels of *Irg1*, but not iNOS (Figures S4G and S4H). *Ifnar1*-deficient macrophages secreted higher levels of IL-1β than WT cells upon inflammasome activation after LPS pre-stimulation (Figure 4I). This effect of IFN signaling is likely mediated in an autocrine manner, because transferring the culture media between WT and *Ifnar1^−/−^* macrophages at 12 h after initial LPS stimulation did not result in tolerization of *Ifnar1^−/−^* cells (Figure S4I). Moreover, even itaconate reconstitution in *Ifnar1^−/−^* macrophages did not rescue inflammasome tolerance (Figure 4I), corroborating the requirement for both IRG1 and iNOS in inflammasome tolerance after LPS pre-treatment. Nonetheless, pre-treatment of *Ifnar1^−/−^* macrophages with 7.5 mM itaconate effectively inhibited classical inflammasome generation (Figure S4J). Itaconate addition is not able to tolerize late NLRP3 inflammasome activation on the *Ifnar1^−/−^* background, yet it inhibits the classical inflammasome, which points to a distinct mechanistic feature between classical and late inflammasome activation.

IFN-I signaling also has a critical role in limiting pro-IL-1β synthesis during LPS stimulation (Guarda et al., 2011). Indeed, we observed that *Ifnar1*-deficient macrophages maintained unusually high levels of pro-IL-1β even at 24 h of LPS stimulation, in contrast to *Nos2^−/−^* or *Irg1^−/−^* macrophages (Figure 4J). Accordingly, *Ifnar1^−/−^* macrophages secreted substantial amounts of IL-1β when triggered with ATP after sustained LPS stimulation (Figure 4K), even without additional LPS restimulation.

### Itaconate regulates pyroptosis after sustained LPS stimulation

**Figure 5.**
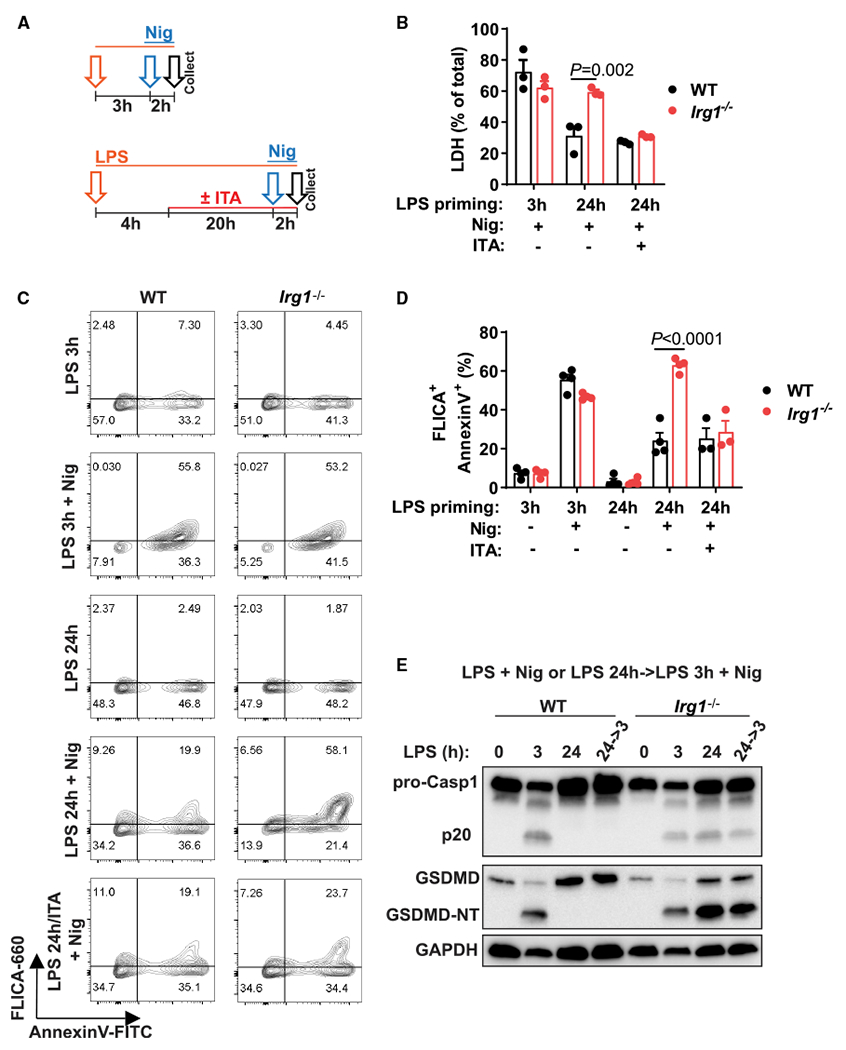
ITA regulates pyroptosis after sustained LPS stimulation (A) Design of classical (3 h of LPS priming) or late (24 h of LPS priming) inflammasome triggering by Nig. In some experiments, ITA was added during the 24 h priming period (ITA, 1 mM, added at 4 h of LPS priming). (B) Lactate dehydrogenase (LDH) activity in supernatants of BMDMs treated as in (A). (C and D) Flow cytometry analysis of pyroptosis in BMDMs treated as in (A). Cell frequencies are shown as percentages. (E) Western blot detection ofcaspase-1 (Casp1)and GSDMD forms in combined cell lysates and supernatants of BMDMs stimulated as in (A). Representative of n = 3 experiments. FLICA, caspase-1-specific fluorochrome inhibitor of caspases. Data are mean ± SEM. In (B) and (E), data are from n = 3–4 experiments. The p values were calculated using 2-way ANOVA with Sidak’s test. See also Figure S5.

In the preceding experiments (Figures 1, 2, 3, and 4), we almost exclusively used the restimulation design (see Figure 1H or 3A) because of the observation that LPS restimulation boosts signal 1 (see RNA-seq data in Figure S1H), which allowed us to observe a robust IL-1β secretion difference between WT and *Irg1^−/−^* cells. However, even late inflammasome triggering alone demonstrated a difference between IL-1β secretion in the two genotypes, albeit to a smaller extent (Figures 1E and 1F). This observation prompted us to consider pro-IL-1β-independent effects of inflammasome activation that can occur without LPS restimulation, e.g., late pyroptosis. Execution of pyroptosis relies largely on the same cellular machinery as IL-1β secretion: GSDMD and caspase-1 are the key effectors of pyroptotic cell death (Liu et al., 2016; Shi et al., 2015). Similar to inflammasome activation, prolonged LPS priming (e.g., 24 h) inhibits pyroptosis upon ATP or nigericin addition. Given that itaconate acts on events associated with signal 2, we hypothesized that *Irg1^−/−^* macrophages might be capable of undergoing late pyroptosis even without secondary restimulation with LPS. Thus, we compared levels of pyroptosis in WT and *Irg1^−/−^* macrophages with LPS priming for 3 or 24 h that was directly followed by nigericin (Figure 5A). The levels of cell death were equivalent for WT and *Irg1^−/−^* macrophages when cells were primed with LPS for 3 h. However, after 24 h of LPS priming, only *Irg1^−/−^* macrophages exhibited high levels of cell death (Figure 5B; Figures S5A and S5B). The excessive death of *Irg1^−/−^* macrophages primed with LPS for 24 h was inhibited when the cells were reconstituted with itaconate during the priming period (Figure 5B). Thus, itaconate acts as an endogenous regulator of pyroptosis under conditions of sustained LPS stimulation.

To confirm that increased cell death results from pyroptosis, we measured caspase-1 activity associated with dying cells. A caspase-1-specific fluorochrome inhibitor of caspases (FLICA) assay in *Irg1^−/−^* macrophages treated with nigericin after 24 h of priming showed substantial caspase-1 activity comparable to that of macrophages primed for 3 h (Figures 5C and 5D). Consistently, caspase-1 activation resulted in release of the p20 fragment of caspase-1; GSDMD was cleaved efficiently in *Irg1^−/−^* cells stimulated with LPS for 24 h, followed by nigericin; and excessive death of *Irg1^−/−^* macrophages was blocked by the caspase-1 inhibitor VX-567 (Figure 5E; Figures S5C and S5D). Collectively, these data reveal a unique regulatory role of itaconate in limiting macrophage pyroptosis upon prolonged LPS stimulation.

### GSDMD, a gatekeeper of late pyroptosis, is itaconated in activated macrophages

**Figure 6.**
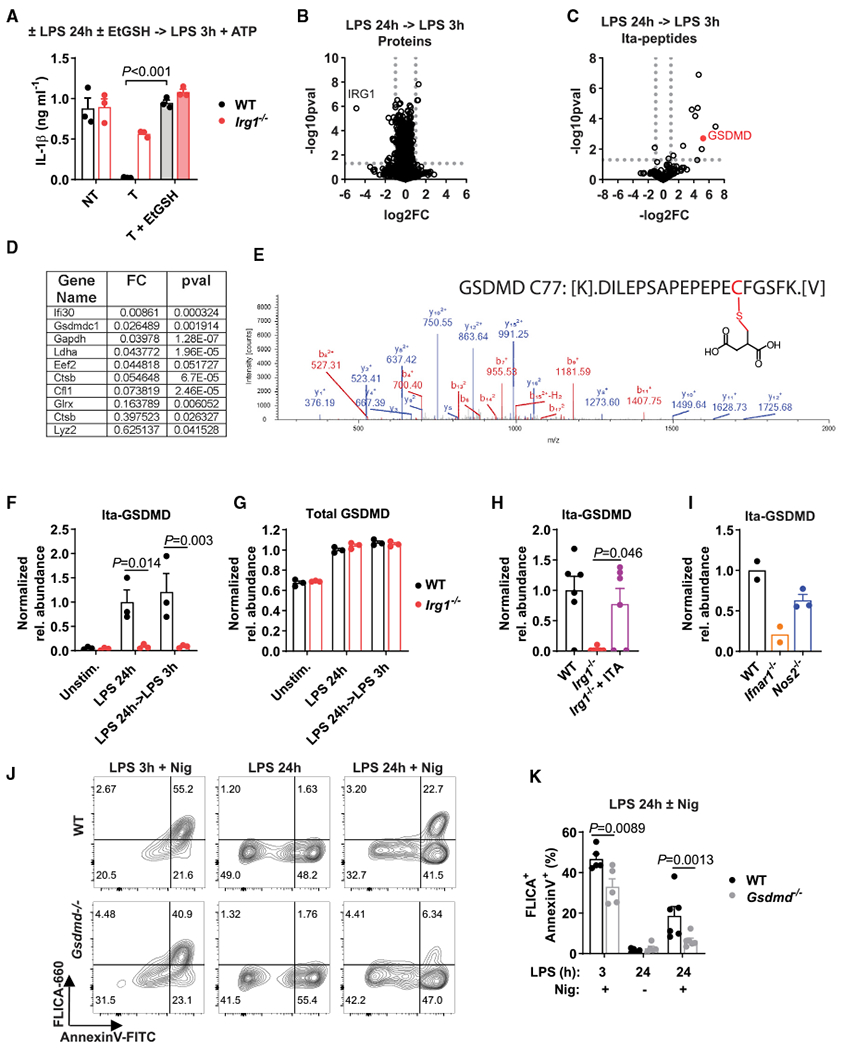
GSDMD is a gatekeeper of late pyroptosis and is itaconated in activated macrophages (A) IL-1β secretion in BMDMs treated as in Figure 1H. EtGSH (5 mM) was added at 4 h of LPS pre-stimulation, with n = 3 experiments. (B and C) Changes in global protein (B) and itaconated-peptide (Ita-peptide; C) content in BMDMs treated as in Figure 1H (with no ATP addition) detected by mass spectrometry, with n = 6, 3 cultures from 2 experiments. The p values were determined with two-tailed, unpaired Student’s t test. (D) Proteins with detected cysteine modification by ITA (itaconation) from (C). (E) Mass spectra and schematics of the structure of the itaconated GSDMD peptide on Cys77. (F–I) Quantification of total GSDMD or itaconated GSDMD on Cys77 (Ita-GSDMD) detected by mass spectrometry (no ATP added). In (H), cells were treated as in Figure 3A (no ATP). In (I), cell were stimulated with LPS for 24 h. In (F) and (G), data are from n = 3 cultures; in (H), n = 6, 3 cultures from 2 experiments; and in (I), n = 2–3 cultures. (J and K) Flow cytometry analysis of pyroptosis in BMDMs treated as in Figure 5A, with n = 5–6 experiments. FC, fold change; pval, p value. Data represent mean ± SEM. pval determined using (A, F, G, and K) 2-way ANOVA with Sidak’s test and (H) 1-way ANOVA with Tukey’s test. See also Figure S6 and Table S2.

We previously showed that itaconate has electrophilic properties and can react with thiol-containing molecules, e.g., glutathione (Bambouskova et al., 2018). It is also known that the addition of cell-permeable glutathione (EtGSH) can mitigate electrophilic and oxidative stress effects (Bambouskova et al., 2018). When we added EtGSH to cells during the LPS pre-stimulation, we observed that WT and *Irg1^−/−^* macrophages produced similarly high amounts of IL-1β even after prolonged priming (Figure 6A). This indicated that electrophilic or oxidative stress can be an important component for establishing late inflammasome tolerance. We have previously shown that electrophilic stress can affect protein levels of important regulators of the macrophage inflammatory response without affecting corresponding transcripts (Bambouskova et al., 2018). Accordingly, we next aimed to explore itaconate-dependent changes in protein expression in primary macrophages. We performed proteomic analysis of macrophages treated with LPS for 24 h or restimulated with LPS and compared protein levels between WT and *Irg1^−/−^* cells. Unexpectedly, the analysis revealed only minor differences in total protein expression (Figure 6B; Figure S6A).

Previously, itaconate-based PTMs were reported in cell lines (Qin et al., 2019) and activated WT primary macrophages at early time points (Mills et al., 2018). Thus, we analyzed our proteomic data with respect to the possible protein modifications by endogenous itaconate. We searched for modifications that are formed through direct covalent bonds between the thiol groups of the cysteine residues within cellular proteins and the electrophilic α,β double bond in the carbon backbone of itaconate. Herein, we specifically refer to protein modifications with unmodified itaconate as itaconation. We were able to detect 10 peptides modified by itaconate on cysteine residues (Figures 6C and 6D; Figure S6B; Table S2), some of which were previously recognized to be modified by itaconate (Mills et al., 2018; Qin et al., 2019). Remarkably, in all tested conditions, one of the strongest signals belonged to the GSDMD peptide, which was not reported previously as a target for endogenous itaconate (Figures 6D and 6E). Itaconated GSDMD was specific to WT (itaconate competent) macrophages at late time points of LPS stimulation (Figure 6F), whereas total levels of GSDMD were unchanged (Figure 6G). This GSDMD modification was also evident upon reconstitution of *Irg1^−/−^* macrophages with 1 mM itaconate (Figure 6H). Activated *Nos2^−/−^* macrophages maintained itaconation of Cys77 in GSDMD, albeit at somewhat lower levels, indicating that a lack of NO does not interfere with the process of GSDMD itaconation. In contrast, this modification is absent from *Ifnar1^−/−^* macrophages, correlating with the lack of tolerance in these cells and suggesting the presence of dedicated IFN-regulated mechanisms responsible for modification of GSDMD by itaconate (Figure 6I). When considering other components of the inflammasome machinery, none of the detected peptides of NLRP3, ASC, or caspase-1 were modified by itaconate. Moreover, because it was shown that itaconate can form itaconyl-coenzyme A (CoA), we searched for protein modification by itaconate in a thioester configuration on either lysine or cysteine residues but were not able to identify either one. However, more labile PTMs might have been missed by the approach that we used for proteomic profiling.

GSDMD processing is considered one of the downstream steps in canonical NLRP3 inflammasome triggering, but the role of GSDMD in late inflammasome activation has not been explored. Thus, we examined whether the role of GSDMD in inflammasome triggering differs between classical and late inflammasomes by using mice genetically deficient in *Gsdmd* (Figure S6C). Because late inflammasome activation of itaconate-competent macrophages does not result in full caspase-1 cleavage to the p20 fragment (see, e.g., Figure 5E), we used a FLICA assay that can determine the catalytic activity of uncleaved caspase-1/pro-caspase-1 species (Boucher et al., 2018). In macrophages primed with LPS for 3 h and then stimulated with nigericin, caspase-1 was activated independently of GSDMD expression, consistent with conventional inflammasome activation reports (Swanson et al., 2019) (Figures 6J and 6K). Late inflammasome activation of WT macrophages demonstrated an increase in caspase-1/pro-caspase-1 activity, indicating that endogenous itaconate interferes with downstream steps. This observation is in line with previous work showing that full caspase-1 processing is not required for cellular caspase-1 activity (Boucher et al., 2018). However, *Gsdmd^−/−^* cells that were LPS primed for 24 h demonstrated nearly complete abrogation of caspase-1 activation based on the FLICA assay (Figures 6J and 6K). Thus, this result establishes that the late NLRP3 inflammasome represents a unique mechanism of triggering by which GSDMD also contributes to the initiation stage of caspase-1/pro-caspase-1 activity.

## DISCUSSION

Our experiments reveal major phenotypes associated with itaconate production by activated macrophages. Our work identifies endogenous itaconate as a regulatory molecule that establishes tolerance to late inflammasome activation and pyroptosis. We identify IRG1 and iNOS as critical co-regulators of late NLRP3 inflammasome tolerance and demonstrate that the ability of different TLR ligands to establish NLRP3 inflammasome tolerance depends on the synergy between IRG1 and iNOS. We detected several post-translational protein modifications by endogenous itaconate and identified GSDMD Cys77 as an itaconation target. These PTMs are found in physiologically relevant settings of prolonged LPS stimulation. Furthermore, the use of *Irg1^−/−^* macrophages as a negative control is critical for identifying PTMs specific to endogenous itaconate. Finally, a distinction needs to be made between profiling of PTMs of itaconate (e.g., Qin et al., 2019) and PTMs of its more electrophilic derivatives, such as octyl-itaconate or similar compounds (e.g., ITalk; Qin et al., 2020). This is important because the electrophilic, metabolic, and immunological properties of these derivatives are different from those of endogenous itaconate (Swain et al., 2020): for instance, Keap1 binding was never directly demonstrated for endogenous or exogenously supplied non-derivatized itaconate, whereas it was observed for octyl-itaconate and its analog ITalk (Mills et al., 2018; Qin et al., 2020). GSDMD has been shown to be a target of cysteine modifications by electrophiles that regulate oligomerization of the GSDMD-NT and pore formation (Hu et al., 2020; Humphries et al., 2020; Qin et al., 2020; Rathkey et al., 2018). It is important to underscore that our work does not establish the functionality of Cys77 modification and that Cys77 has been shown previously to be dispensable for the oligomerization process (Liu et al., 2016). However, given that the impact of itaconate is already evident on the level of GSDMD processing and early caspase-1 activation events, we hypothesize that Cys77 itaconation might be involved in the interplay between GSDMD and caspase-1.

Our work continues a line of investigation focused on endogenous itaconate or non-derivatized itaconate (Bambouskova et al., 2018; Cordes et al., 2016; Lampropoulou et al., 2016; Shen et al., 2017; Swain et al., 2020). This is in contrast to studies that consider itaconate derivatives as suitable proxies to study endogenous itaconate’s properties. Because striking differences between the immunological and the electrophilic properties of itaconate and itaconate derivatives (octyl-itaconate, dimethyl-itaconate, etc.) have been established (Bambouskova et al., 2018; Swain et al., 2020), the continued indiscriminate usage of the term “itaconate” with respect to data obtained through utilization of its more electrophilic derivatives introduces confusion to the field (e.g., see Hooftman et al., 2020; Humphries et al., 2020; Mills et al., 2018). For instance, work by Hooftman et al. (2020) used octyl-itaconate to probe the mild inflammasome phenotype that was reported in Lampropoulou et al. (2016) and reproduced the data from Swain et al. (2020). Experiments by Hooftman et al. (2020) detected cysteine modification of the NLRP3 protein by octyl-itaconate in the context of the classical (early) inflammasome. However, neither Hooftman et al. (2020) nor our work detected cysteine modifications of the NLRP3 protein by endogenous/non-derivatized itaconate. Finally, our mechanistic data suggest that endogenous itaconate affects processes downstream of ASC, rather than directly affecting NLRP3. Specifically, our experiments demonstrate that prolonged stimulation of WT macrophages tolerizes inflammasome activation at the level of caspase-1 processing: caspase-1/pro-caspase-1 activity detected by FLICA is observed in tolerized WT macrophages (after 24 h of LPS), yet it does not result in detectable caspase-1 p20 fragment cleavage. This establishes that the initiation of caspase-1 activity can occur in the presence of itaconate but does not proceed into the stage that allows for its cleavage. We found that the itaconate-mediated tolerance to inflammasome activation is specific to canonical NLRP3 activation. This is surprising, because different inflammasomes, such as AIM2, also rely on the function of caspase-1 and GSDMD. We speculate that this might be a consequence of specific requirements of NLRP3 activation, such as potassium efflux, molecular assembly, or cellular localization. The basis of specificity toward NLRP3 remains an open question and warrants further investigation.

Furthermore, although attention has been devoted recently to the interplay between TCA cycle remodeling and inflammatory regulation, our data demonstrate that the major phenotype of inflammasome tolerance by itaconate is decoupled from its TCA regulation at the level of SDH activity. We find that neither SDH inhibition nor succinate supplementation can regulate the levels of IL-1β secretion. This observation is not in contrast with the Tannahill et al. (2013) report of an IL-1β-succinate connection, because that work used a distinct succinate derivative and did not demonstrate that endogenous succinate is a regulator of IL-1β secretion in macrophages.

Lastly, an important aspect of our work is the mechanistic difference between early and late inflammasomes. Several key observations establish this difference. First, we show differential involvement of GSDMD between the two types of inflammasomes. In contrast to the conventional early inflammasome settings, we find that in the late inflammasome initial caspase-1 activation steps are GSDMD dependent. We hypothesize that in the environment of late NLRP3 inflammasome triggering, activation of GSDMD by initial active caspase-1/pro-caspase-1 species leads to amplification of the signal needed for full caspase-1 activation and effective substrate processing. Second, we demonstrate that the late inflammasome has different redox dependence compared with the conventional early inflammasome: supplementing media with EtGSH rescues IL-1 β secretion during the late inflammasome, whereas adding antioxidants to the conventional inflammasome leads to inhibition of IL-1 β secretion, e.g., because of ROS inhibition (Dostert et al., 2008; Iyer et al., 2013; Tschopp and Schroder, 2010). We believe this observation reflects redox-dependent changes occurring during prolonged LPS activation that lead to inflammasome tolerance. Third, although exogenously added non-derivatized itaconate can inhibit both early (conventional) and late inflammasome activation, only the early inflammasome requires the persistent presence of itaconate in the media. This is evident because washing out exogenous itaconate from the cell media before restimulation still leads to inhibition of inflammasome activation, whereas this same wash before the early inflammasome abrogates activation (Figure S4A). Mechanistically, this might be explained by different patterns of PTMs by itaconate in inflammatory versus resting macrophages (although this is not the only possible explanation). Altogether, we argue that late inflammasome activation in *Irg1^−/−^* is an important phenotype distinct from conventional inflammasome activation and that it is the most relevant *in vitro* experimental design to study the physiological importance and regulatory mechanisms of endogenous itaconate.

Overall, our work reveals the physiological function of itaconate as a key tolerance molecule. Future studies are warranted to define the complete molecular mechanism of late NLRP3 inflammasome triggering and the specific role of itaconate in its inhibition.

## STAR★METHODS

### RESOURCE AVAILABILITY

#### Lead contact

Further information and requests for resources and reagents should be directed to and will be fulfilled by the Lead Contact, Maxim N. Artyomov (martyomov@wustl.edu).

#### Materials availability

This study did not generate new unique reagents.

#### Data and code availability

The mass spectrometry proteomics data have been deposited to the ProteomeXchange Consortium via the PRIDE partner repository with the dataset identifier PXD020089. The raw and processed RNA-seq data have been deposited in the Gene Expression Omnibus with accession number GEO: GSE164383. Source data for metabolomics in Figures 3 and S3 is available in Table S1.

### EXPERIMENTAL MODEL AND SUBJECT DETAILS

#### Experimental animals

*Irg1^−/−^* C57BL/6N mice were described previously (Lampropoulou et al., 2016). C57BL/6N used as WT controls in experiments with *Irg1^−/−^* mice were obtained from Charles River Laboratories. B6.129X1-*Nfe2l2^tm1Ywk^*/J (*Nrf2^−/−^*), C57BL/6N-*Gsdmd^em4Fcw^*/J (*Gsdmd^−/−^*), B6.Cg-Gt(ROSA)26Sor^tm1(CAG-Pycard/mCitrine*,-CD2*)Dtg^/J (ASC-mCitrine (Tzeng et al., 2016), B6.129P2-*Nos2^tm1Lau^*/J (*Nos2^−/−^*), B6(Cg)-*Ifnar1^tm.2Ees^*/J (*Ifnar1^−/−^*) and WT C57BL/6J mouse strains were purchased from Jackson Laboratory. ROSA26-CreER^T2^/SDHB^floxed/floxed^ (*Sdhb*^fl/fl^) and ROSA26-CreER^T2^/SDHB^wild-type/wild-type^ mice (*Sdhb* WT) as previously published (Cardaci et al., 2015) were provided by Eyal Gottlieb (University of Glasgow, UK). Mice were maintained at Washington University under specific pathogen-free conditions in accordance with Federal and University guidelines and protocols approved by the Animal Studies Committee of Washington University. Femurs and tibias from *Atf3^−/−^* mice as described (Jiang et al., 2004) were provided by Tsonwin Hai (Ohio State University). Mice used for *in vitro* experiments were 8- to 24-week-old, age and sex-matched and both sexes were used. For *in vivo* experiments 9 to 14-week-old female mice were used in the model of endotoxin-induced sepsis and 10 to 12-week-old male mice were used in the psoriasis model.

#### Human samples

The Washington University in St. Louis Institutional Review Board reviewed and approved a study for the collection of blood samples from healthy subjects (IRB approval #201804084). Written consent was obtained from all participants. Donors represented one male and two females, 35-50 years old.

### METHOD DETAILS

#### Bone marrow-derived macrophages (BMDMs)

BMDM were prepared from 8- to 24-week-old mice. Bone marrow cells were isolated from femurs and tibias and cultured in RPMI-1640 medium supplemented with 10% heat-inactivated fetal bovine serum (FBS; Hyclone), 2 mM L-glutamine, and 100 U/mL penicillin-streptomycin (complete media; cRPMI) and mouse recombinant M-CSF (20 ng/mL, Peprotech). Fresh cRPMI containing M-CSF was added at day 4 post isolation. At the day 7 post isolation cells were non-enzymatically harvested using Cell Stripper (GIBCO) and seeded in cRPMI at concentration 8 × 10^5^-10^6^ cells/mL in tissue culture plates of various formats.

*Sdhb*^fl/fl^ and *Sdhb* WT BMDMs were given a first dose of tamoxifen (600 nM, Sigma) at day 4 of differentiation and a second dose at the time of plating for experiment together with one extra dose of M-CSF. Cells then were rested for 24h before stimulation.

#### BMDM stimulations

For NLRP3 inflammasome stimulations lipopolysaccharide (LPS; 100 ng/mL; from *E. coli* 0111:B4, Sigma) was added for intervals as indicated and followed by addition of either ATP (5 mM, adenosine 5′-triphosphate disodium salt Sigma) or nigericin (Nig, 5 μM; InvivoGen), time depending on the procedure. For tolerization, cells were stimulated or not with LPS for 24h, then media was changed to fresh cRPMI and cells were incubated for 1h without stimulant. After this period, a second round of LPS was added and incubated for 3 h, followed by ATP addition. In some experiments, different TLR stimulants were used for pre-stimulation: poly(I:C) (PIC, 20 μg/mL; InvivoGen), Pam3CSK4 (PCSK, 200 ng/mL; InvivoGen) or their combination. In some experiments, itaconic acid, malonic acid or succinic acid were used for pre-treatment or added at 4 h of pre-stimulation with LPS and then washed out before re-stimulation. All metabolites stock solutions were prepared as 250 mM, in tissue culture-grade water. In tolerization and late inflammasome activation protocols, itaconate was added at 4h of LPS pre-stimulation and 1 mM itaconate was used if not stated otherwise. Caspase inhibitors: zVAD-fmk (zVAD; pan-caspase inhibitor, Selleckchem), Ac-YVAD-cmk (YVAD; caspase-1 inhibitor, Sigma), VX-765 (caspase-1 inhibitor, Selleckchem), zIETD-fmk (IETD; caspase-8 inhibitor, BioVision) all 50 μM if not indicated otherwise, were added 30 min before stimulation with ATP. GSH ethyl ester (EtGSH; 5 mM, Sigma) was added in tolerization protocol at 4h of LPS pre-stimulation and then washed out before re-stimulation. In experiments with MCC950 (20 μM, InvivoGen) the drug was added 30 min before nigericin. In some experiments S-ethylisothiourea (SEIT, Cayman) was added at 1 h of LPS pre-stimulation, 500 μM was used if not stated otherwise. S-Nitroso-N-acetyl-DL-penicillamine (SNAP, Sigma) was at 4 h of LPS pre-stimulation. L-Buthionine-sulfoximine (BSO, 500 μM, Sigma) was added simultaneously with LPS stimulation. ForAIM2 stimulations poly(dA:dT) (PAT; InvivoGen) was first incubated with Lipofectamine 2000 (LPF; Invitrogen) for 10 min in OptiMEM (GIBCO) at room temperature. Mixture was then added to cells to final concentration 0.5 μg/ml PAT for 6 h. For non-canonical NLRP3 activation LPS was mixed with LPF and the mixture was added to cells to final concentration of LPS 100 ng/ml for 18h.

#### Human monocyte-derived macrophages (MoDMs)

Venous blood was collected in Sodium-Heparin vacutainers (Cat# 367874, BD). Plasma and blood cells were separated using Histopaque-1077 (Cat# 10771, Sigma) according to the manufacturer’s protocol. Briefly, whole blood was diluted 1:1 with sterile DPBS-2 mM EDTA (Sigma or Corning) and overlaid (30 mL) on to 10 mL of Histopaque-1077. Gradients were centrifuged at 500xg for 30 min. The peripheral blood mononuclear cell (PBMC) layer at the plasma-Histopaque interface was transferred to a new tube and washed twice with cold DBPS-EDTA. PBMCs were isolated and CD16+ cells depleted using anti-human CD16 magnetic beads (Cat# 130-045-701, Miltenyi) using manufacturer’s protocol. After CD16 depletion, CD14+ monocytes were purified using anti-human CD14 magnetic beads (Cat# 130-050-201, Miltenyi). Monocytes were plated in 24-well tissue culture treated plates at density 5x10^5^ per well in 1 mL of cRPMI supplemented with human recombinant M-CSF (50 ng/ml; Peprotech). Every two days the media was exchanges for fresh cRPMI containing M-CSF. At the day 7-8 the media was changed to cRPMI without M-CSF and stimulations were carried out as indicated. MoDMs were stimulated with LPS (100 ng/ml) and ATP (5 mM, 45 min).

#### *In vivo* model of endotoxin-induced sepsis

Sodium itaconate (Na-ITA) was prepared by adding 1:2010 M NaOH (Sigma, Cat# 72068) to 250 mM stock solution of itaconic acid in PBS. Female WT C57BL/6 mice were treated intraperitoneally (i. p.) with sodium itaconate (Na-ITA, 1.24 mg/mouse in PBS) or vehicle control (PBS) for 20 h and 4 h before intraperitoneal stimulation with LPS (4 mg kg^−1^; Sigma). 6 h later mice were euthanized, and peritoneal fluid samples were collected.

#### Western blots

In experiments where protein in supernatants were analyzed, BMDMs were plated in cRPMI and then at the time of LPS addition media was changed to OptiMEM (Cat# 31985-070, Thermo Fisher). In tolerization experiments, the first stimulation was performed in cRPMI and media was switched to OptiMEM after 24h. ATP or nigericin was added for duration as indicated. Cells were lysed in RIPA Lysis Buffer System (SCBT). Cell supernatants were precipitated with methanol/cholorophorm and resuspended in RIPA Lysis Buffer System. Lysates were heat-denatured at 95°Cfor5 min in reducing Laemmli Sample Buffer (BioRad). Proteins were separated on 4%-20% polyacrylamide gradient gels (BioRad) and transferred onto 0.45 mm pore size PVDF membranes (Millipore). Non-specific binding was blocked with 5% skim milk (w/v) or 1% skim milk (w/v) when proteins in supernatants were analyzed and membranes were probed with following primary antibodies: IL-1β (#12507), cleaved IL-1β (#52718S), iNOS (#13120) from CellSignaling; caspase-1 (cl. Casper-1, #AG-20B-0042), NLRP3 (#AG-20B-0014) from Adipogen; GSDMD (#ab209845), SDHB (#ab14714) from Abcam and, GAPDH (#sc-32233). Secondary HRP-conjugated antibodies and proteins: mouse IgGk-binding protein (#sc-516102) and mouse anti-rabbit-IgG (#sc-2357) from SCBT. Membranes were incubated with Clarity western ECL substrate (BioRad) and imaged with ChemiDoc Imaging System (BioRad). GAPDH run on the same blot was used as loading control. Bands were cropped and densitometry performed using ImageJ (Schneider et al., 2012). Uncropped, unprocessed western blots with size marker are provided in Data S1.

#### NO measurements

BMDMs in cRPMI were activated with 100 ng/ml LPS for 24 h. After one hour of LPS activation SEIT was added to a final concentration of 250 μM, 500 μM and 1000 μM, respectively. Nitrite levels were determined in the cell supernatants using Griess Reagent System (Promega) according to manufacturer’s instructions.

#### RNA isolation, real time quantitative PCR and used primers

RNA from cultured cells was isolated using a E.Z.N.A.® Total RNA Kit I (Omega). Isolated RNA was reverse-transcribed using Random Primers (Cat# 48190-011, Invitrogen) and AffinityScript Multi-Temp Reverse Transcriptase (Agilent) according to the manufacturer’s protocol. Reactions were performed using a SYBR Green PCR Master mix (Thermo Fisher Scientific) and a LightCycler 480 (Roche Diagnostics). All assays were performed at least in duplicate, and reaction mixtures 10-μl volumes (384-plate) were processed under the following cycling conditions: initial 10 min denaturation at 95 °C, followed by 40 cycles at 95 °C for 10 s, 60 °C for 1 min. Threshold cycle values for each sample were determined by automated threshold analysis. Expression levels of all mRNAs were normalized to reference gene *Actb* in BMDMs. The relative increase in the expression level of a gene was normalized to the level of expression in unstimulated control WT cells in each experiment.

#### Cytokine detection by ELISA

ATP or nigericin was added for 45 min. Cytokines in cell supernatants or peritoneal fluid were analyzed using DuoSet® ELISA (mouse IL-1β, mouse IL-6, mouse TNF, human IL-1β R&D Systems) according to manufacturer protocol. Mouse IL-18 was detected by IL-18 Mouse ELISA Kit (Invitrogen). For IL-1β and IL-18 assays supernatants from cells were diluted 1:2, for IL-6 and TNF 1:4.

#### RNA sequencing

mRNA was extracted from cell pellets using oligo-dT beads (Invitrogen). Custom oligo-dT primers containing barcode and adaptor-linker sequences (CCTACACGACGCTCTTCCGATCT-XXXXXXXX-T15) were used to for cDNA synthesis. Barcoded first-strand synthesis cDNAs for each sample were pooled together, and the RNA-DNA hybrid was degraded by acid-alkali treatment. A second sequencing adaptor (AGATCGGAAGAGCACACGTCTG) was ligated using T4 ligase (NEB) followed by SPRI bead cleanup (Beckman-Coulter). The libraries were PCR amplified 12 cycles, followed by SPRI bead clean-up prior to sequencing. RNA-seq libraries were sequenced at The Centre for Applied Genomics at the Hospital for Sick Children in Toronto on a HiSeq 2500 using 50 bp × 25 bp paired-end sequencing. Sequences for each sample were aligned to mm10 using the STAR aligner. All RNA-seq experiments were performed in n = 3 independent cultures. Heatmaps were generated using the Phantasus online service (https://artyomovlab.wustl.edu/phantasus/).

#### Caspase activity *in vitro*

To assess the activity of caspase-1, we used human recombinant caspase-1 (Abcam). The enzyme (0.2U) was incubated with sodium itaconate (Na-ITA; prepared by adding 1:20 10 M NaOH (Sigma) to 250 mM stock solution of itaconic acid) at 37°C for 30 min in reaction buffer (HEPES 50 mM, NaCl 200 mM, KCl 50 mM, DTT 10 mM, Digitonin 100 μg/mL, pH = 8). Then, Ac-YVAD-afc substrate (100 μM, SCBT) was added to samples and kinetic measurements of fluorescence (405/510 nm) were taken every minute for 1h using a Synergy H1 plate reader (BioTek). The final fluorescence at 1 h was used for calculation of relative activity.

#### ASC specking

In itaconate pre-treatment experiments BMDMs derived from ACS-mCitrine mice were used. Cells were harvested after 1 h of nigericin stimulation and resuspended in PBS. In experiments using WT and *Irg1^−/−^* BMDMs, cells were stimulated as indicated, fixed in 2% paraformaldehyde for 15 min and permeabilized with 0.5% Triton X-100 for 10 min. Subsequently cells were incubated with PE-conjugated anti-ASC antibody (1:100; BioLegend) overnight in 0.5% BSA in PBS (w/v), washed 3x with PBS and analyzed. Samples were acquired using BD CantoII and data were analyzed using FlowJo software. Results were acquired with the FACSDiva software (BD) and specking cells were determined based on scattering properties of labeled mCitrine or PE fluorescence (Hoss et al., 2018) as PE-H high and PE-W low population using FlowJo software (Tree Star).

#### Cytotoxicity assay

BMDMs were plated in cRPMI and media was changed to OptiMEM when LPS was added. ATP or nigericin was added for 2 h. Lactate dehydrogenase (LDH) activity in cell supernatants was measured with CytoTOX 96 (Promega) according to manufacturer’s instructions. Cell lysates used as control for total LDH content were prepared for each treatment/genotype condition.

#### Pyroptosis measurements by flow cytometry

BMDMs were loaded with FLICA 660 Caspase-1 Assay (final dilution of the FLICA 660 reagent for incubation with cells was 1:300, ImmunoChemistry) after 1h of treatment with nigericin and incubated for additional 1h. Cells then were washed twice with washing buffer and harvested using Cell Stripper (GIBCO). Collected cells were centrifuged and resuspended in annexin binding buffer (10 mM HEPES, 0.14 M NaCl, 2.5 mM CaCl_2_, pH 7.4) containing AnnexinV-FITC (Biolegend) and LIFE/DEAD Aqua dead cell stain (Thermo Fisher) and incubated for 15 min. Cells were diluted 1:3 with annexin binding buffer and acquired with a BD CantoII. Results were acquired with the FACSDiva software (BD) and FLICA^+^AnnexinV^+^ population was quantified using FlowJo software (Tree Star).

#### *In vivo* psoriasis model

Imiquimod cream (IMQ, 5%, Perrigo) was applied daily to both ears (8 mg/ear) for 10 days. On day 11, mice were euthanized, and ears were processed for cell isolation (1/2 from each ear). For cell isolation, ears were cut into 4x4 mm pieces and digested for 90 min at 37°C in the presence of DNase I (100 μg/mL, Sigma), Liberase TL (85 μg/mL, Roche) and hyaluronidase (60 Units/mL, Sigma), dissolved in DMEM supplemented with 2% heat-inactivated FBS, HEPES pH 7.3 (final concentration of 10 mM) and 100 U/mL penicillin-streptomycin. Single cell suspensions were prepared by homogenizing softened tissue with a GentleMACS dissociator (Miltenyi). Ear-draining lymph nodes also were collected, and single-cell suspensions generated by mashing lymph nodes through a 70 μm filter. Single cells from both the ear and the lymph node were re-stimulated with PMA (10 ng/mL, Millipore Sigma) and ionomycin (500 ng/mL, Life Technologies) in the presence of Brefeldin A (1x, Thermo Fisher) for 4h to assess cytokine production. Cells were harvested and stained with Aqua LIVE/DEAD fixable dye (Invitrogen) and fluorochrome conjugated antibodies to surface CD45-BUV563 (cl. 30-F11, #612924) and TCRd-BV650 (cl. GL3, #563993) were from BD Biosciences; CD3-AF488 (cl. 145-2C11, #100321), CD4-BV711 (cl. GK1.5, #100549), CD8-BV421 (cl. 53-6., #100738), CD11b-PE/Cy7 (cl. M170, #101216), MHCII-PE (cl. M5/114.15.2, #107607), Ly6C-APC/Cy7 (cl. HK.4, #128025), Ly6G-BV605 (cl. 1A8, #127639) were from Biolegend; and CD11c-AF700 (cl. N418, #56-0114-82) from Invitrogen. Intracellular cytokines were detected using the BD cytofix/cytoperm kit (BD Biosciences) according to manufacturer’s instructions with mouse IL-17A-APC-Cy7 (cl. TC11.18H10.1, #506922, Biolegend). Stained cells were acquired on a BD Symphony A3. Results were acquired with the FACSDiva software (BD) and analyzed using FlowJo software (Tree Star). Gating strategy is shown in Data S2.

#### Mass spectrometry metabolic analysis

Cells were rapidly washed twice with ice cold 0.9% NaCl and snap frozen on dry ice. Metabolites were extracted with 1mL of ice-cold extraction solvent (40% acetonitrile, 40% methanol, 20% water (v/v)), and the metabolite containing supernatant was dried in a speedvac. Dried extracts were resuspended in 50 μL of water for LC/MS analysis as described previously (Roy et al., 2020). Briefly, analysis was completed using ion-paired reversed phase liquid chromatography using an HPLC (1290 Infinity II, Agilent Technologies, Santa Clara, CA) coupled to a triple quadrupole mass spectrometer (6470, Agilent) with electrospray ionization operated in negative mode. The column was a ZORBAX Rapid Resolution HD (2.1 × 150 mm, 1.8 μm pore size; #759700-902, Agilent). Mobile phase A was 3% methanol (in H_2_O) and mobile phase B was 100% methanol. Both mobile phases contained 10mM of the ion-pairing agent tributylamine (#90780, SigmaAldrich, St. Louis, MO, USA), 15mM acetic acid, and 2.5 μM medronic acid (#5191-4506, Agilent Technologies, Santa Clara, CA, USA). The LC gradient was: 0-2.5min 100%A, 2.5-7.5 min ramp to 80%A, 7.5-13min ramp to 55%A, 13-20min ramp to 99%B, 20-24 min hold at 99%B. Flow rate was 0.25 mL/min, and the column compartment heated to 35°C. The column was then backflushed with 100% acetonitrile for 4 minutes (ramp from 0.25 to 0.8mL/min in 1.5 minutes) and re-equilibrated with mobile phase A for 5 minutes at 0.4 mL/min. Two itaconate transitions (m/z: 129 → 85, 129 → 41) were monitored at RT = 13.6 min ± 1 min. For itaconate quantitation, serial half-log dilutions were prepared from a chemical standard (#I29204, Sigma) from 1 mM to 0.32 nM, of which 1 mL was dried and resuspended in 50 μL of water. These were analyzed immediately after the biological samples from the lowest to highest concentration and regression analysis of standards was used to quantitate itaconate levels in biological samples using MassHunter Quantitative Analysis Software (v9.0, Agilent).

Carboxylates such as citrate, the precursor to itaconate, are better detected and quantitated in GC/MS analysis. In order to address this, following LC/MS analysis, the samples were dried using a speedvac and stored at −80°C overnight. Trimethylsilyl derivatives were prepared from biological samples and itaconate standards. Briefly, samples were resuspended in 30 μl of pyridine (#PX2012-7, Sigma) with methoxyamine (10 mg/mL w/v; #89803, Sigma), vortexed for 5 minutes, incubated at 70°C for 20 minutes, and cooled to room temperature. Then, 70μl of N-Methyl-N-(trimethylsilyl)trifluoroacetamide with 1% trimethylchlorosilane (MSTFA + 0.1% TMCS; #69478, Sigma) was added. The samples were peulse vortexed and incubated for 90 minutes, with an additional pulse vortex every 30 minutes. GC/MS analysis was conducted using a GC/MSD (Agilent 7890B GC/5977B MSD, Agilent Technologies, Santa Clara, CA) equipped with a J&W DB-5ms GC Column (30 m, 0.25 mm, 0.25 mm with DuraGuard, 10 m, Agilent). The MS was equipped with a high efficiency source (HES) operating in electron impact (EI) mode set at −70eV. The autosampler (Agilent 7693A Autosampler, Agilent) collected 1 μl of sample and injected the sample at a 10:1 split using helium as the carrier gas at a flow rate of 1 ml/min. The inlet temperature was set at 320°C. The initial oven temperature was 95°C held for 1 minute. The oven was then ramped to 118°C at a rate of 40°C/min and held at 118°C for 2 min. Next the oven was ramped to 250°C at a rate of 12°C/min and then ramped to 320°C at a rate of 40°C/min and held for 7 minutes. Sample data was acquired by scanning from 50 to 800 m/z. The response for itaconate was determined using MassHunter Quantitative Analysis Software (v9.0, Agilent) at a retention time of 7.58 ± 0.1 min, quantifier ion m/z: 259, and qualifier ions m/z: 215, 230, and 133. Relative metabolite abundance data from both LC/MS and GC/MS platforms are provided in Table S1.

#### Mass spectrometry proteomics

In brief, cell pellets were lysed in 8M urea and cysteines were reduced and alkylated. Proteins were sequentially digested with LysC (1:50 enzyme:substrate, Wako Chemicals) and trypsin (1:100 enzyme:substrate, Promega) and desalted by solid phase extraction. Peptide amounts were normalized and labeled with TMT10plex Isobaric Label Reagents (Thermo Fisher). Pooled peptides were fractionated by basic pH reverse phase into a 96-well plate, and fractions were consolidated into 24 samples in a checkerboard manner. Peptides were separated on a 50 μM C18 EASY-Spray column (Thermo Scientific) using a 70 min, 8–28% acetonitrile gradient, and spectra were acquired on an Oribtrap Fusion (Thermo Scientific) using a TMT-MS3 method. All RAW files were processed using Proteome Discoverer 2.2 and searched against the Uniprot mouse reference proteome (UP000000589). Searches were conducted with dynamic modifications allowed for methionine oxidation (+15.995), lysine itaconylation (+112.016), cysteine itaconation (+130.026) or cysteine carbamidomethylation (+57.021). Signal-to-noise values for all peptides were summed within each TMT channel, and each channel was scaled according to the highest channel sum so that the sum abundance of each channel is equal. Peptides were filtered for a minimum sum signal-to-noise value of 160 across all 10 channels. Quantitative data from razor peptides were excluded and only unique peptides were used for protein quantitation.

### QUANTIFICATION AND STATISTICAL ANALYSIS

Unless stated otherwise in a specific section of STAR methods, standard statistical analyses were performed using Prism Software v8.3.0 (Graph Pad). The type and number of replicates and the statistical test used are described in the figure legends. Exact *P values* are shown where determined. Individual data points are shown, and the mean ± standard error of the mean (SEM) is reported for analyses with n ≥ 3.

## Supplementary Material

1

2

## Figures and Tables

**Table:** KEY RESOURCES TABLE

REAGENT or RESOURCE	SOURCE	IDENTIFIER
**Antibodies**		
IL-1β, rabbit monoclonal Ab	CST	Cat# 12507; **RRID: AB_2721117**
cleaved IL-1β (Asp117), rabbit polyclonal Ab	CST	Cat# 52718; **RRID:AB_2799421**
iNOS, rabbit monoclonal Ab	CST	Cat# 13120; **RRID:AB_2687529**
Caspase-1, mouse monoclonal Ab (cl. Casper-1)	AdipoGen	Cat# AG-20B-0042; **RRID:AB_2490248**
NLRP3/NALP3, mouse monoclonal Ab (cl. Cryo-2)	AdipoGen	Cat# AG-20B-0014; **RRID:AB_2490202**
GSDMD, rabbit monoclonal Ab	Abcam	Cat# ab209845; **RRID:AB_2783550**
SDHB, mouse monoclonal Ab	Abcam	Cat# ab14714; **RRID:AB_301432**
GAPDH, mouse monoclonal Ab	SCBT	Cat# sc-32233; **RRID:AB_627679**
mouse anti-rabbit-IgG-HRP	SCBT	Cat# sc-2357; **RRID:AB_628497**
ASC, PE, mouse monoclonal Ab	Biolegend	Cat# 653904; **RRID:AB_2564508**
CD3, AF488, hamster monoclonal Ab	Biolegend	Cat# 100321; **RRID:AB_389300**
CD4, BV711, rat monoclonal Ab	Biolegend	Cat# 100549; **RRID:AB_11219396**
CD8, BV421, rat monoclonal Ab	Biolegend	Cat# 100738; **RRID:AB_11204079**
CD11b, PE/Cy7, rat monoclonal Ab	Biolegend	Cat# 101216; **RRID:AB_312799**
MHCII, PE, rat monoclonal Ab	Biolegend	Cat# 107607; **RRID:AB_313322**
Ly6C, APC/Cy7, rat monoclonal Ab	Biolegend	Cat# 128025; **RRID:AB_10643867**
Ly6G, BV605, rat monoclonal Ab	Biolegend	Cat# 127639; **RRID:AB_2565880**
IL-17A, APC-Cy7, rat monoclonal Ab	Biolegend	Cat# 506922; **RRID:AB_2125010**
CD11c, AF700, hamster monoclonal Ab	Invitrogen	Cat# 56-0114-82; **RRID:AB_493992**
CD45, BUV563, rat monoclonal Ab	BD	Cat# 612924; **RRID:AB_2870209**
TCRd, BV650, hamster monoclonal Ab	BD	Cat# 563993; **RRID:AB_2738530**
**Biological samples**		
Human PBMCs	This paper	N/A
Chemicals, peptides, and recombinant proteins		
M-CSF, murine recombinant	Peprotech	315-02
M-CSF, human recombinant	Peprotech	300-25
mouse IgG_κ_-binding protein, HRP	SCBT	sc-516102
Fetal bovine serum characterized, heat inactivated	Hyclone	SH30071.03
Tamoxifen	Sigma	SML1666
LPS from *E.coli* 0111:B4	Sigma	L4391
Adenosine 5′-triphosphate disodium salt	Sigma	A6419
Nigericin	InvivoGen	#tlr-nig
Poly(I:C) HMW	InvivoGen	#tlrl-pic
Pam3CSK4	InvivoGen	#tlrl-pms
Itaconic acid	Sigma	I29204
Malonic acid	Sigma	M1296
Succinic acid	Sigma	S9512
zVAD-fmk	Selleckchem	S7023
Ac-YVAD-cmk	Sigma	SML0429
VX-765	Selleckchem	S2228
zEITD-fmk	SCBT	sc-3084
Glutathione ethyl ester	Sigma	G1404
MCC950	InvivoGen	Inh-mcc
S-ethylisothiourea	Cayman	81275
S-Nitroso-N-acetyl-DL-penicillamine	Sigma	N3398
L-Buthionine-sulfoximine	Sigma	B2515
Poly(dA:dT)	InvivoGen	tlr-patn
Lipofectamine 2000	Invitrogen	11668027
NaOH 10 M BioUltra	Sigma	72068
RIPA Lysis Buffer System	SCBT	sc-24948
AffinityScript Multi-Temp reverse transcriptase	Agilent	600109
SYBR Green PCR Master mix	Thermo Fisher	4309155
Digitonin	Sigma	D141
Ac-YVAD-afc substrate	SCBT	sc-311282
AnnexinV-FITC	Biolegend	640906
LIFE/DEAD Aqua dead cell stain	ThermoFisher	L34957
Imiquimod cream	Perrigo	N/A
DNase I	Sigma	11284932001
Liberase TL	Sigma	5401020001
Hyaluronidase	Sigma	389561
**Critical commercial assays**		
Griess Reagent System	Promega	Cat# G2930
CytoTOX 96	Promega	Cat# G1780
IL-1β Mouse DuoSet ELISA	R&D	Cat# DY401
IL-6 Mouse DuoSet ELISA	R&D	Cat# DY406
TNF Mouse DuoSet ELISA	R&D	Cat# DY410
IL-1β Human DuoSet ELISA	R&D	Cat# DY201
IL-18 Mouse ELISA Kit	Invitrogen	Cat# BMS618-3
BD Cytofix/Cytoperm Kit	BD	Cat# 554714
FLICA 660 Caspase 1 Assay	ImmunoChemistry	Cat# 9122
E.Z.N.A.® Total RNA Kit I	Omega	Cat# R6834-01
Deposited data		
Raw and analyzed RNA-Seq data	This paper	GEO: GSE164383
Mass spectrometry proteomics data	This paper	PRIDE: PXD020089
Mass spectrometry metabolomics data	This paper	Table S1
Mass spectrometry data for itaconate levels in Figure 1D	Swain et al., 2020	N/A
**Experimental models: organisms/strains**		
Mouse: *Irg1^−/−^* C57BL/6N	Lampropoulou et al., 2016	N/A
Mouse: C57BL/6NCrl	Charles River Laboratory	Stock# 027
Mouse: *Nrf2^−/−^*: B6.129X1-*Nfe212^tm1Ywk^*/J	The Jackson Laboratory	Stock# 017009
Mouse: *Gsdmd^−/−^*: *C57BL/6N-Gsdmd^em4Fcw^*/J	The Jackson Laboratory	Stock# 032410
Mouse: ASC-mCitrine: B6.Cg-Gt(ROSA)26Sor^tm1(CAG-Pycard/mCitrine*,-CD2*)Dtg^/J	The Jackson Laboratory	Stock# 030744
Mouse: *Nos2^−/−^*: B6.129P2-*Nos2^tm1Lau^*/J	The Jackson Laboratory	Stock# 002609
Mouse: *Ifnar1^−/−^*: B6(Cg)-*Ifnar1^tm.2Ees^*/J	The Jackson Laboratory	Stock# 028288
Mouse: C57BL/6J	The Jackson Laboratory	Stock# 000664
Mouse: *Sdhb*^fl/fl^: ROSA26-CreER^T2^/SDHB^floxed/floxed^	Cardaci et al., 2015	N/A
Mouse: *Sdhb* WT: ROSA26-CreER^T2^/SDHB^wildtype/wildtype^	Cardaci et al., 2015	N/A
Mouse: *Atf3^−/−^*	Jiang et al., 2004	N/A
**Oligonucleotides**		
Mouse Actb forward primer 5- > 3: GGAGGGGGTTGAGGTGTT	IDT	N/A
Mouse Actb reverse primer 5- > 3: TGTGCACTTTTATTGGTCTCAAG	IDT	N/A
Mouse Irg1 forward primer 5- > 3: GTTTGGGGTCGACCAGACTT	IDT	N/A
Mouse Irg1 reverse primer 5- > 3: CAGGTCGAGGCCAGAAAACT	IDT	N/A
**Software and algorithms**		
Phantasus	Developed by Sergushichev team at ITMO University	https://ctlab.itmo.ru/phantasus/
ImageJ v1.52p	Schneider et al., 2012	Schneider et al., 2012
FlowJo V 10.6	Tree Star	https://www.flowjo.com/solutions/flowjo/downloads
MassHunter Quantitative Analysis Software V9.0	Agilent	https://www.agilent.com/en/product/software-informatics/mass-spectrometry-software/data-analysis/quantitative-analysis
Prism V 8.3.0	GraphPad Software	https://www.graphpad.com/scientific-software/prism/
FACSDiva V 7.0 software	BD	https://www.bdbiosciences.com/en-us/instruments/research-instruments/research-software/flow-cytometry-acquisition/facsdiva-software
Biorender	Biorender	https://biorender.com/
Proteome Discoverer V 2.2	Thermo Fisher	https://www.thermofisher.com/us/en/home/industrial/mass-spectrometry/liquid-chromatography-mass-spectrometry-lc-ms/lc-ms-software/multi-omics-data-analysis/proteome-discoverer-software.html

## Highlights

- Itaconate tolerizes macrophages to late NLRP3 inflammasome (i.e., long LPS priming)
- Itaconate acts downstream of ASC, affecting caspase-1/GSDMD interplay
- Late inflammasome require GSDMD for caspase-1 activation; GSDMD-Cys77 is itaconated
- iNOS and IRG1 jointly establish late inflammasome tolerance

## References

[R1] Bailey JD, Diotallevi M, Nicol T, McNeill E, Shaw A, Chuaiphichai S, Hale A, Starr A, Nandi M, Stylianou E, (2019). Nitric Oxide Modulates Metabolic Remodeling in Inflammatory Macrophages through TCA Cycle Regulation and Itaconate Accumulation. Cell Rep. 28, 218–230.e7. 31269442 10.1016/j.celrep.2019.06.018 PMC6616861

[R2] Bambouskova M, Gorvel L, Lampropoulou V, Sergushichev A, Loginicheva E, Johnson K, Korenfeld D, Mathyer ME, Kim H, Huang L-H, (2018). Electrophilic properties of itaconate and derivatives regulate the IκBζ-ATF3 inflammatory axis. Nature 556, 501–504. 29670287 10.1038/s41586-018-0052-z PMC6037913

[R3] Boucher D, Monteleone M, Coll RC, Chen KW, Ross CM, Teo JL, Gomez GA, Holley CL, Bierschenk D, Stacey KJ, (2018). Caspase-1 self-cleavage is an intrinsic mechanism to terminate inflammasome activity. J. Exp. Med 215, 827–840. 29432122 10.1084/jem.20172222 PMC5839769

[R4] Cardaci S, Zheng L, MacKay G, van den Broek NJF, MacKenzie ED, Nixon C, Stevenson D, Tumanov S, Bulusu V, Kamphorst JJ, (2015). Pyruvate carboxylation enables growth of SDH-deficient cells by supporting aspartate biosynthesis. Nat. Cell Biol 17, 1317–1326. 26302408 10.1038/ncb3233 PMC4591470

[R5] Coll RC, Robertson AAB, Chae JJ, Higgins SC, Muñoz-Planillo R, Inserra MC, Vetter I, Dungan LS, Monks BG, Stutz A, (2015). A small-molecule inhibitor of the NLRP3 inflammasome for the treatment of inflammatory diseases. Nat. Med 21, 248–255. 25686105 10.1038/nm.3806 PMC4392179

[R6] Cordes T, Wallace M, Michelucci A, Divakaruni AS, Sapcariu SC, Sousa C, Koseki H, Cabrales P, Murphy AN, Hiller K, and Metallo CM (2016). Immunoresponsive Gene 1 and Itaconate Inhibit Succinate Dehydrogenase to Modulate Intracellular Succinate Levels. J. Biol. Chem 291, 14274–14284. 27189937 10.1074/jbc.M115.685792 PMC4933182

[R7] Dostert C, Pétrilli V, Van Bruggen R, Steele C, Mossman BT, and Tschopp J (2008). Innate immune activation through Nalp3 inflammasome sensing of asbestos and silica. Science 320, 674–677. 18403674 10.1126/science.1156995 PMC2396588

[R8] Farlik M, Reutterer B, Schindler C, Greten F, Vogl C, Müller M, and Decker T (2010). Nonconventional initiation complex assembly by STAT and NF-kappaB transcription factors regulates nitric oxide synthase expression. Immunity 33, 25–34. 20637660 10.1016/j.immuni.2010.07.001 PMC2914224

[R9] Foster SL, Hargreaves DC, and Medzhitov R (2007). Gene-specific control of inflammation by TLR-induced chromatin modifications. Nature 447, 972–978. 17538624 10.1038/nature05836

[R10] Guarda G, Braun M, Staehli F, Tardivel A, Mattmann C, Förster I, Farlik M, Decker T, Du Pasquier RA, Romero P, and Tschopp J (2011). Type I interferon inhibits interleukin-1 production and inflammasome activation. Immunity 34, 213–223. 21349431 10.1016/j.immuni.2011.02.006

[R11] Hernandez-Cuellar E, Tsuchiya K, Hara H, Fang R, Sakai S, Kawamura I, Akira S, and Mitsuyama M (2012). Cutting edge: nitric oxide inhibits the NLRP3 inflammasome. J. Immunol 189, 5113–5117. 23100513 10.4049/jimmunol.1202479

[R12] Hirotani T, Yamamoto M, Kumagai Y, Uematsu S, Kawase I, Takeuchi O, and Akira S (2005). Regulation of lipopolysaccharide-inducible genes by MyD88 and Toll/IL-1 domain containing adaptor inducing IFN-beta. Biochem. Biophys. Res. Commun 328, 383–392. 15694359 10.1016/j.bbrc.2004.12.184

[R13] Hooftman A, Angiari S, Hester S, Corcoran SE, Runtsch MC, Ling C, Ruzek MC, Slivka PF, McGettrick AF, Banahan K, (2020). The Immunomodulatory Metabolite Itaconate Modifies NLRP3 and Inhibits Inflammasome Activation. Cell Metab. 32, 468–478.e7. 32791101 10.1016/j.cmet.2020.07.016 PMC7422798

[R14] Hoss F, Rolfes V, Davanso MR, Braga TT, and Franklin BS (2018). Detection of ASC Speck Formation by Flow Cytometry and Chemical Cross-linking BT. Methods Mol. Biol 1714, 149–165. 29177861 10.1007/978-1-4939-7519-8_10

[R15] Hu JJ, Liu X, Xia S, Zhang Z, Zhang Y, Zhao J, Ruan J, Luo X, Lou X, Bai Y, (2020). FDA-approved disulfiram inhibits pyroptosis by blocking gasdermin D pore formation. Nat. Immunol 21, 736–745. 32367036 10.1038/s41590-020-0669-6 PMC7316630

[R16] Humphries F, Shmuel-Galia L, Ketelut-Carneiro N, Li S, Wang B, Nemmara VV, Wilson R, Jiang Z, Khalighinejad F, Muneeruddin K, (2020). Succination inactivates gasdermin D and blocks pyroptosis. Science 369, 1633–1637. 32820063 10.1126/science.abb9818 PMC8744141

[R17] Iyer SS, He Q, Janczy JR, Elliott EI, Zhong Z, Olivier AK, Sadler JJ, Knepper-Adrian V, Han R, Qiao L, (2013). Mitochondrial cardiolipin is required for Nlrp3 inflammasome activation. Immunity 39, 311–323. 23954133 10.1016/j.immuni.2013.08.001 PMC3779285

[R18] Jha AK, Huang SC-C, Sergushichev A, Lampropoulou V, Ivanova Y, Loginicheva E, Chmielewski K, Stewart KM, Ashall J, Everts B, (2015). Network integration of parallel metabolic and transcriptional data reveals metabolic modules that regulate macrophage polarization. Immunity 42, 419–430. 25786174 10.1016/j.immuni.2015.02.005

[R19] Jiang H-Y, Wek SA, McGrath BC, Lu D, Hai T, Harding HP, Wang X, Ron D, Cavener DR, and Wek RC (2004). Activating transcription factor 3 is integral to the eukaryotic initiation factor 2 kinase stress response. Mol. Cell. Biol 24, 1365–1377. 14729979 10.1128/MCB.24.3.1365-1377.2004 PMC321431

[R20] Lampropoulou V, Sergushichev A, Bambouskova M, Nair S, Vincent EEE, Loginicheva E, Cervantes-Barragan L, Ma X, Huang SC-CCC, Griss T, (2016). Itaconate Links Inhibition of Succinate Dehydrogenase with Macrophage Metabolic Remodeling and Regulation of Inflammation. Cell Metab. 24, 158–166. 27374498 10.1016/j.cmet.2016.06.004 PMC5108454

[R21] Liu X, Zhang Z, Ruan J, Pan Y, Magupalli VG, Wu H, and Lieberman J (2016). Inflammasome-activated gasdermin D causes pyroptosis by forming membrane pores. Nature 535, 153–158. 27383986 10.1038/nature18629 PMC5539988

[R22] Mao K, Chen S, Chen M, Ma Y, Wang Y, Huang B, He Z, Zeng Y, Hu Y, Sun S, (2013). Nitric oxide suppresses NLRP3 inflammasome activation and protects against LPS-induced septic shock. Cell Res. 23, 201–212. 23318584 10.1038/cr.2013.6 PMC3567828

[R23] McGinley AM, Sutton CE, Edwards SC, Leane CM, DeCourcey J, Teijeiro A, Hamilton JA, Boon L, Djouder N, and Mills KHG (2020). Interleukin-17A Serves a Priming Role in Autoimmunity by Recruiting IL-1β-Producing Myeloid Cells that Promote Pathogenic T Cells. Immunity 52, 342–356.e6. 32023490 10.1016/j.immuni.2020.01.002

[R24] Medzhitov R, Schneider DS, and Soares MP (2012). Disease tolerance as a defense strategy. Science 335, 936–941. 22363001 10.1126/science.1214935 PMC3564547

[R25] Michelucci A, Cordes T, Ghelfi J, Pailot A, Reiling N, Goldmann O, Binz T, Wegner A, Tallam A, Rausell A, (2013). Immune-responsive gene 1 protein links metabolism to immunity by catalyzing itaconic acid production. Proc. Natl. Acad. Sci. USA 110, 7820–7825. 23610393 10.1073/pnas.1218599110 PMC3651434

[R26] Mills EL, Ryan DG, Prag HA, Dikovskaya D, Menon D, Zaslona Z, Jedrychowski MP, Costa ASH, Higgins M, Hams E, (2018). Itaconate is an anti-inflammatory metabolite that activates Nrf2 via alkylation of KEAP1. Nature 556, 113–117. 29590092 10.1038/nature25986 PMC6047741

[R27] Mishra BB, Rathinam VAK, Martens GW, Martinot AJ, Kornfeld H, Fitzgerald KA, and Sassetti CM (2013). Nitric oxide controls the immunopathology of tuberculosis by inhibiting NLRP3 inflammasome-dependent processing of IL-1 β. Nat. Immunol 14, 52–60. 23160153 10.1038/ni.2474 PMC3721324

[R28] Qin W, Qin K, Zhang Y, Jia W, Chen Y, Cheng B, Peng L, Chen N, Liu Y, Zhou W, (2019). S-glycosylation-based cysteine profiling reveals regulation of glycolysis by itaconate. Nat. Chem. Biol 15, 983–991. 31332308 10.1038/s41589-019-0323-5

[R29] Qin W, Zhang Y, Tang H, Liu D, Chen Y, Liu Y, and Wang C (2020). Chemoproteomic Profiling of Itaconation by Bioorthogonal Probes in Inflammatory Macrophages. J. Am. Chem. Soc 142, 10894–10898. 32496768 10.1021/jacs.9b11962

[R30] Rathkey JK, Zhao J, Liu Z, Chen Y, Yang J, Kondolf HC, Benson BL, Chirieleison SM, Huang AY, Dubyak GR, (2018). Chemical disruption of the pyroptotic pore-forming protein gasdermin D inhibits inflammatory cell death and sepsis. Sci. Immunol 3, eaat2738. 30143556 10.1126/sciimmunol.aat2738 PMC6462819

[R31] Roy DG, Chen J, Mamane V, Ma EH, Muhire BM, Sheldon RD, Shorstova T, Koning R, Johnson RM, Esaulova E, (2020). Methionine Metabolism Shapes T Helper Cell Responses through Regulation of Epigenetic Reprogramming. Cell Metab. 31, 250–266.e9. 32023446 10.1016/j.cmet.2020.01.006

[R32] Schneider CA, Rasband WS, and Eliceiri KW (2012). NIH Image to ImageJ: 25 years of image analysis. Nat. Methods 9, 671–675. 22930834 10.1038/nmeth.2089 PMC5554542

[R33] Shen H, Campanello GC, Flicker D, Grabarek Z, Hu J, Luo C, Banerjee R, and Mootha VK (2017). The Human Knockout Gene CLYBL Connects Itaconate to Vitamin B_12_. Cell 171, 771–782.e11. 29056341 10.1016/j.cell.2017.09.051 PMC5827971

[R34] Shi J, Zhao Y, Wang K, Shi X, Wang Y, Huang H, Zhuang Y, Cai T, Wang F, and Shao F (2015). Cleavage of GSDMD by inflammatory caspases determines pyroptotic cell death. Nature 526, 660–665. 26375003 10.1038/nature15514

[R35] Swain A, Bambouskova M, Kim H, Andhey PS, Duncan D, Auclair K, Chubukov V, Simons DM, Roddy TP, Stewart KM, and Artyomov MN (2020). Comparative evaluation of itaconate and its derivatives reveals divergent inflammasome and type I interferon regulation in macrophages. Nat. Metab 2, 594–602. 32694786 10.1038/s42255-020-0210-0 PMC7378276

[R36] Swanson KV, Deng M, and Ting JP-Y (2019). The NLRP3 inflammasome: molecular activation and regulation to therapeutics. Nat. Rev. Immunol 19, 477–489. 31036962 10.1038/s41577-019-0165-0 PMC7807242

[R37] Tannahill GM, Curtis AM, Adamik J, Palsson-McDermott EM, McGettrick AF, Goel G, Frezza C, Bernard NJ, Kelly B, Foley NH, (2013). Succinate is an inflammatory signal that induces IL-1β through HIF-1α. Nature 496, 238–242. 23535595 10.1038/nature11986 PMC4031686

[R38] Tschopp J, and Schroder K (2010). NLRP3 inflammasome activation: The convergence of multiple signalling pathways on ROS production? Nat. Rev. Immunol 10, 210–215. 20168318 10.1038/nri2725

[R39] Tzeng T-C, Schattgen S, Monks B, Wang D, Cerny A, Latz E, Fitzgerald K, and Golenbock DT (2016). A Fluorescent Reporter Mouse for Inflammasome Assembly Demonstrates an Important Role for Cell-Bound and Free ASC Specks during In Vivo Infection. Cell Rep. 16, 571–582. 27346360 10.1016/j.celrep.2016.06.011 PMC5384574

