# Supplementary material for: Itaconate confers tolerance to late NLRP3 inflammasome activation
Source: Cell Rep. Author manuscript; Available in PMC 2021 Apr 12. (PMC8039864; doi:10.1016/j.celrep.2021.108756)
Supplement: 1 [file NIHMS1683088-supplement-1.pdf]

Cell Reports, Volume *34*

Supplemental information

Itaconate confers tolerance to late NLRP3 inflammasome activation

Monika Bambouskova, Lucie Potuckova, Tomas Paulenda, Martina Kerndl, Denis A. Mogilenko, Kate Lizotte, Amanda Swain, Sebastian Hayes, Ryan D. Sheldon, Hyeryun Kim, Unnati Kapadnis, Abigail E. Ellis, Christine Isaguirre, Samantha Burdess, Anwesha Laha, Gaya K. Amarasinghe, Victor Chubukov, Thomas P. Roddy, Michael S. Diamond, Russell G. Jones, Donald M. Simons, and Maxim N. Artyomov

Figure S1

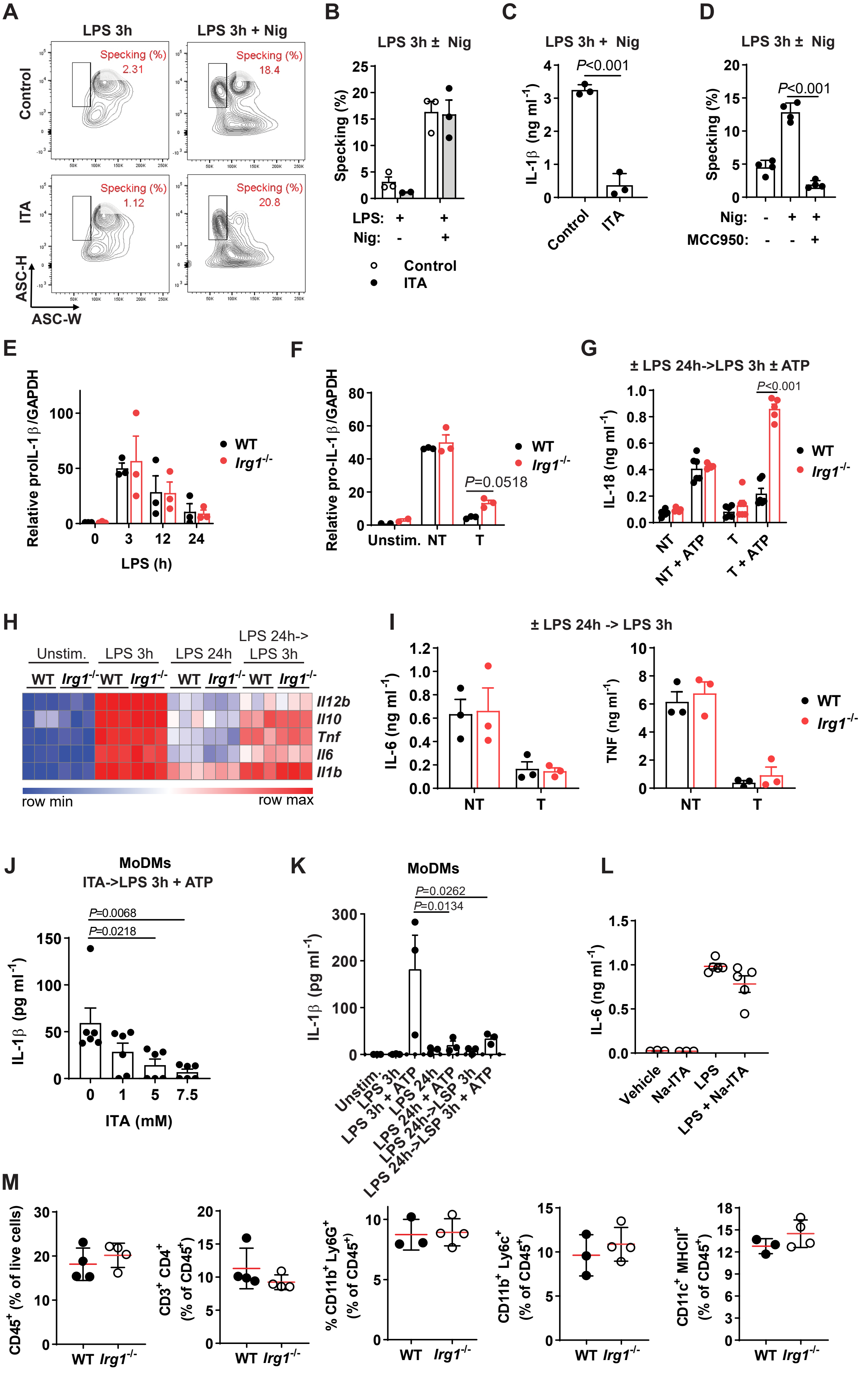

A
LPS 3h
LPS 3h + Nig
Control
ITA
Specking (%) 2.31
Specking (%) 18.4
Specking (%) 1.12
Specking (%) 20.8
ASC-H
ASC-W
B
LPS 3h ± Nig
Specking (%)
LPS:
Nig:
Control
ITA
C
LPS 3h + Nig
P<0.001
IL-1β (ng ml-1)
Control
ITA
D
LPS 3h ± Nig
P<0.001
Specking (%)
Nig:
MCC950:
E
Relative proIL-1β/GAPDH
LPS (h)
WT
Irg1-/-
F
Relative pro-IL-1β/GAPDH
P=0.0518
Unstim.
NT
T
WT
Irg1-/-
G
± LPS 24h->LPS 3h ± ATP
P<0.001
IL-18 (ng ml-1)
NT
NT + ATP
T
T + ATP
WT
Irg1-/-
H
Unstim.
LPS 3h
LPS 24h
LPS 24h-> LPS 3h
WT
Irg1-/-
Il12b
Il10
Tnf
Il6
Il1b
row min
row max
I
± LPS 24h -> LPS 3h
IL-6 (ng ml-1)
TNF (ng ml-1)
NT
T
WT
Irg1-/-
J
MoDMs
ITA->LPS 3h + ATP
P=0.0068
P=0.0218
IL-1β (pg ml-1)
ITA (mM)
K
MoDMs
P=0.0262
P=0.0134
IL-1β (pg ml-1)
Unstim.
LPS 3h
LPS 3h + ATP
LPS 24h
LPS 24h + ATP
LPS 24h->LSP 3h
LPS 24h->LSP 3h + ATP
L
IL-6 (ng ml-1)
Vehicle
Na-ITA
LPS
LPS + Na-ITA
M
CD45+ (% of live cells)
CD3+ CD4+ (% of CD45+)
% CD11b+ Ly6G+ (% of CD45+)
CD11b+ Ly6c+ (% of CD45+)
CD11c+ MHCII+ (% of CD45+)
WT
Irg1-/-

Figure S1. *Irg1* establishes LPS-mediated tolerance to signal 2 of NLRP3 inflammasome activation. Related to Figure 1. (**A**) ASC-mCitrine expressing BMDMs were pretreated with 7.5 mM itaconate (ITA) for 3 h and stimulated with LPS for 3 h followed by nigericin (Nig) for 1 h. ASC specking was determined by flow cytometry. (**B**) Quantification of results in (A), n = 3 experiments. (**C**) IL-1β detection in supernatants of ASC-mCitrine BMDMs treated and stimulated as in (A), n = 3 experiments. (**D**) ASC-mCitrine expressing BMDMs were stimulated with LPS for 3 h followed by nigericin (Nig) for 1 h. MCC950 was added 30 min before Nig. ASC specking was determined by flow cytometry. n = 4 experiments. (**E**, **F**) Densitometric analysis of western blots from Figure 1G and J. n = 3 experiments. (**G**-**I**) Cytokine analysis in BMDMs stimulated with LPS as determined by RNA-Seq (H) or in supernatants (G), (I). T, tolerized; NT, non-tolerized. In (G) n = 4; 2 cultures from 2 experiments. In (H) n = 3 cultures. In (I) n = 3 experiments. (**J**) IL-1β secretion in human monocyte-derived macrophages (MoDMs) treated and stimulated as in Figure 1A. n = 6; 3 cultures from 2 donors. (**K**) IL-1β secretion in MoDM stimulated as indicated, n = 3 donors. (**L**) Cytokine analysis in peritoneal lavage of mice challenged as in Figure 1K. (n = 3 vehicle, Na-ITA; n = 5 LPS, Na-ITA + LPS). (**M**) Cell frequencies in ear tissue determined by flow cytometry. Mice were treated with IMQ as described in Figure 1L, n = 3-4 mice per group. Data represent mean ± SEM. *P* values were determined using (B), (F), (G) 2-way ANOVA with Sidak's test; (C), (M) unpaired two-tailed Student's t-test; (D), (J), (K), (L) 1-way ANOVA with Tuckey's test.

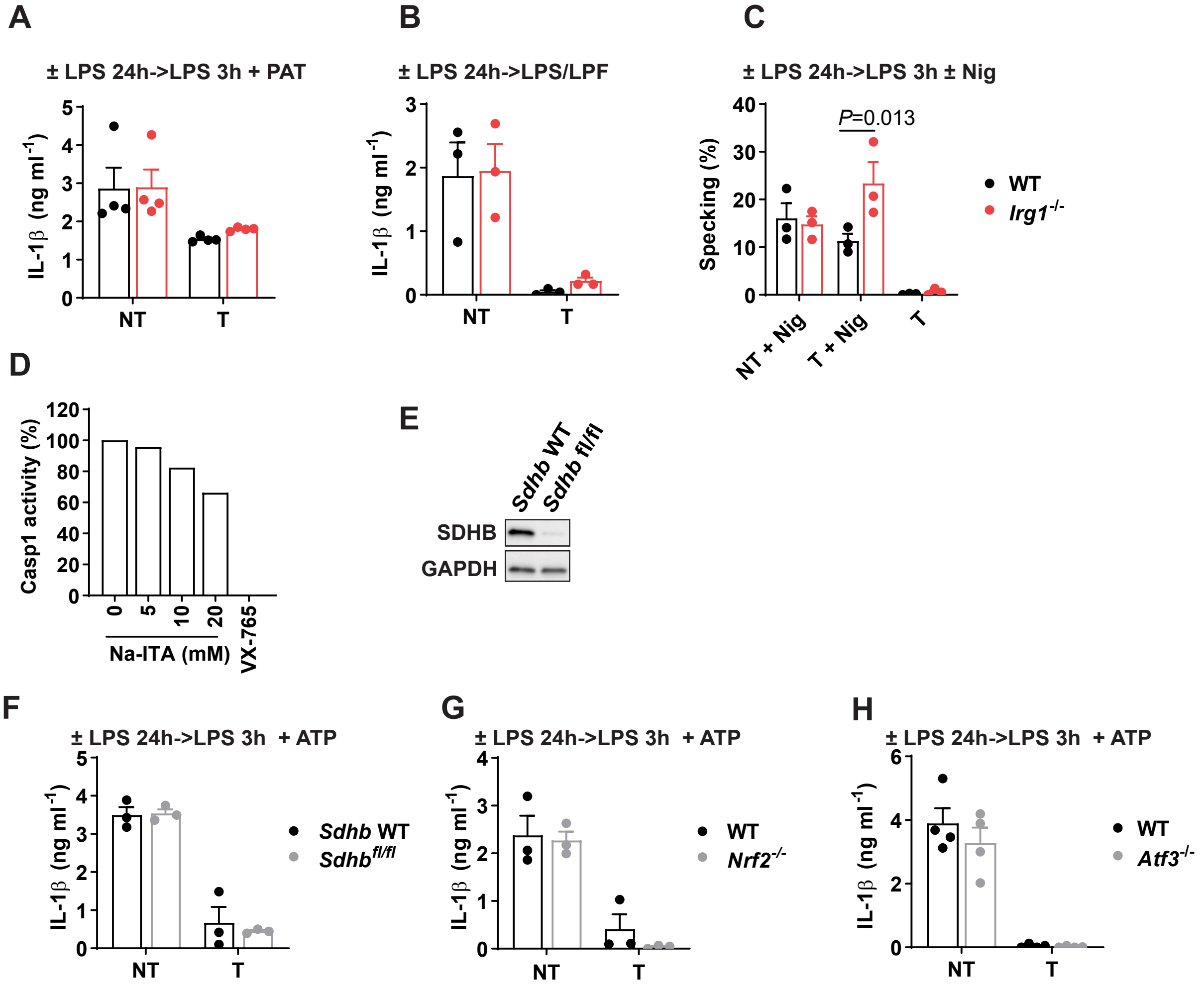

Figure S2. *Irg1* phenotype is associated with dysregulation of caspase-1 and GSDMD processing. Related to Figure 2. (**A**) IL-1β secretion in BMDMs stimulated as in Figure 1H except instead of ATP, the cells were stimulated with poly(dA:dT) for 6h (PAT). (**B**) IL-1β secretion in BMDMs stimulated as in Figure 1H except instead of LPS 3h + ATP, the cells were stimulated with LPS in the presence of lipofectamine (LPF) for 18h. (**C**) Quantification of the flow cytometry data from Figure 2A, n = 3 experiments. (**D**) Enzymatic activity of recombinant caspase-1 p20 in the presence of sodium itaconate (Na-ITA) or VX-765 (caspase-1 inhibitor, 50 μM). Data are mean of n = 2 replicates. (**E**) SDHB expression analyzed in tamoxifen treated BMDMs. GAPDH was used as loading control. (**F**-**H**) IL-1β secretion in the cell supernatants of BMDMs of indicated genotypes stimulated as in Figure 1H, n=3-4 experiments. Data represent mean + SEM. *P* values were calculated using 2-way ANOVA with Sidak's test.

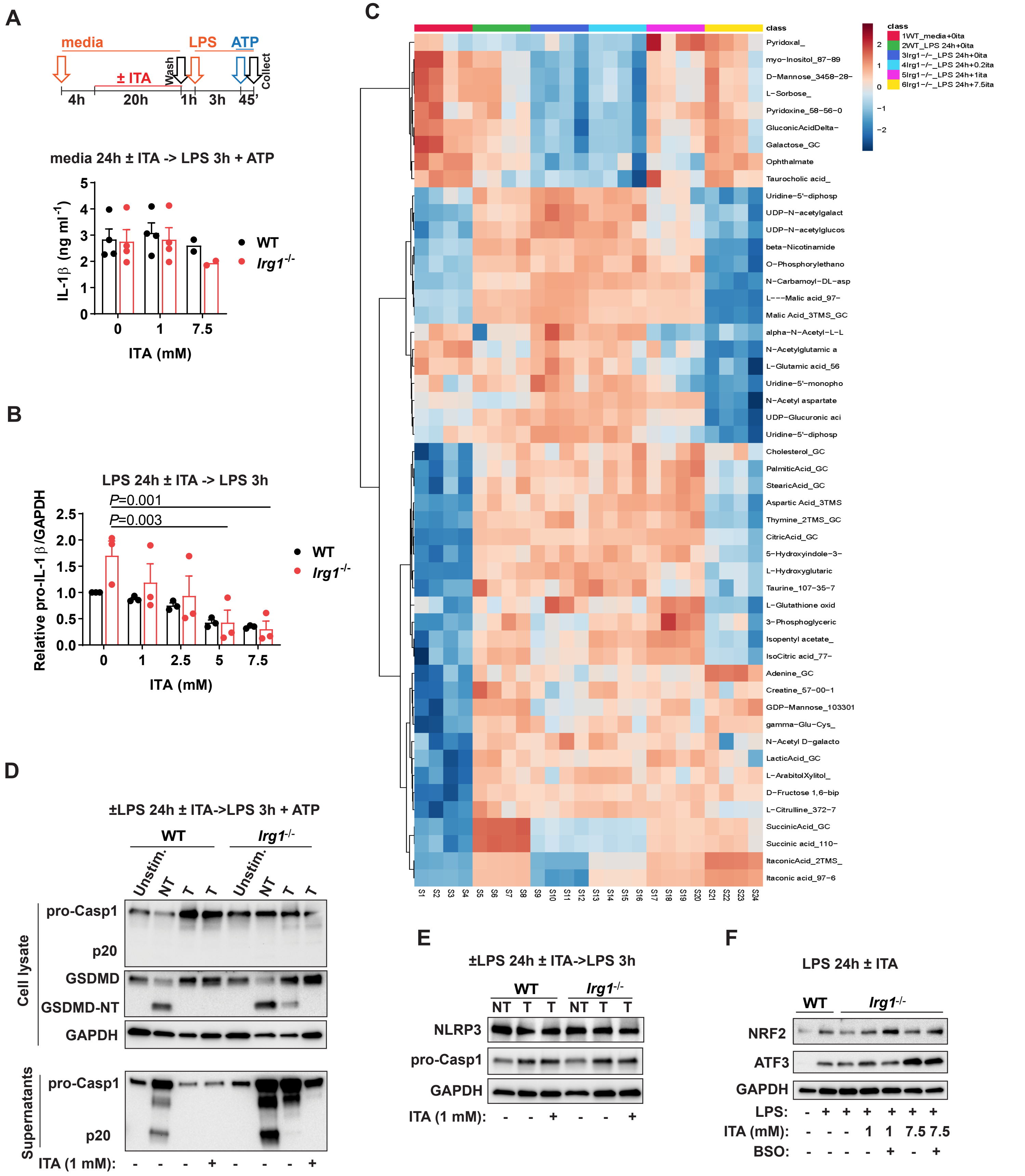

Figure S3. Itaconate reconstitution rescues inflammasome tolerance in *Irg1-/-*. Related to Figure 3. (**A**) IL-1β detection in supernatants of BMDMs treated and stimulated as shown by the schematics, n = 2-4 experiments. (**B**) Densitometric analysis of western blots from Figure 3C, n = 3 experiments. Data represent mean ± SEM, *P* values were calculated using 2-way ANOVA, Tuckey's test. (**C**) Top 50 differentially expressed metabolites by ANOVA, scale bar represents row z-score. Metabolites from both LC/MS and GC/MS platforms are shown. (**D**) Western blot detection of proteins in lysates and supernatants of BMDMs stimulated and treated as in Figure 3A. NT, non-tolerized; T, tolerized; Unstim., unstimulated control. (**E**) Western blot detection of proteins in lysates of BMDMs stimulated and as in Figure 3A, no ATP was added. (**F**) Protein detection in lysates of BMDMs stimulated with LPS for 24h. BSO (500 µM) was added simultaneously with LPS. Itaconate (ITA) as indicated was added at 4h of LPS stimulation. All WB data are representative of n=3 experiments, GAPDH was used as loading control.

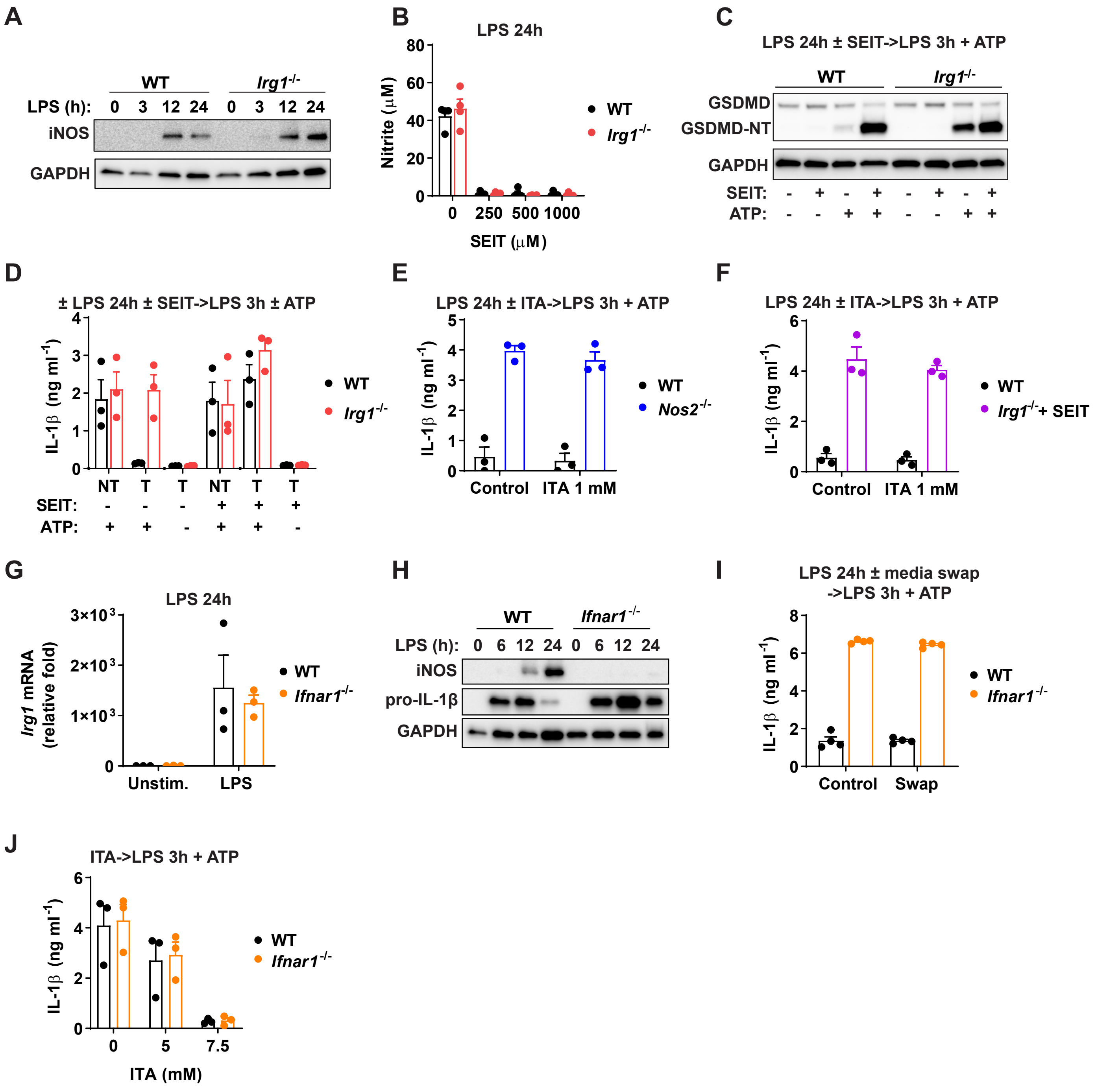

Figure S4. Itaconate synergizes with iNOS to tolerize NLRP3 inflammasome activation. Related to Figure 4. (**A**) iNOS detection in lysates of BMDMs stimulated with LPS for indicated time. (**B**) Nitrite detection in supernatant of BMDMs stimulated with LPS for 24 h, SEIT was added at 1 h of LPS stimulation, n = 4 experiments. (**C**) GSDMD detection in cell lysates of BMDMs treated as in Figure 1H. SEIT (500 μM) was added at 1 h of LPS pre-stimulation. (**D**) IL-1β secretion in cell supernatants of BMDMs stimulated and treated as in Figure 3A. SEIT (500 μM) was added at 1 h of LPS pre-stimulation. (**E**, **F**) IL-1β secretion in cell supernatants of BMDMs stimulated and treated as in Figure 3A. SEIT (500 μM) was added at 1 h of LPS pre-stimulation, n = 3 experiments. (**G**) Irg1 mRNA expression in BMDMs stimulated with LPS for 24h, n = 3 experiemtns. (**H**) Protein expression in lysates of BMDMs stimulated with LPS for indicated time. (**I**) IL-1β secretion in BMDMs stimulated as in Figure 1H. Cell media were swapped between the genotypes at 12 h of LPS pre-stimulation period, n = 4 cultures. (**J**) IL-1β secretion in BMDMs treated with itaconate (ITA) and stimulated with LPS and ATP as in Figure 1A, n = 4 experiments. Data represent mean + SEM. Western blots are representative of n = 3 experiments, GAPDH was used as loading control.

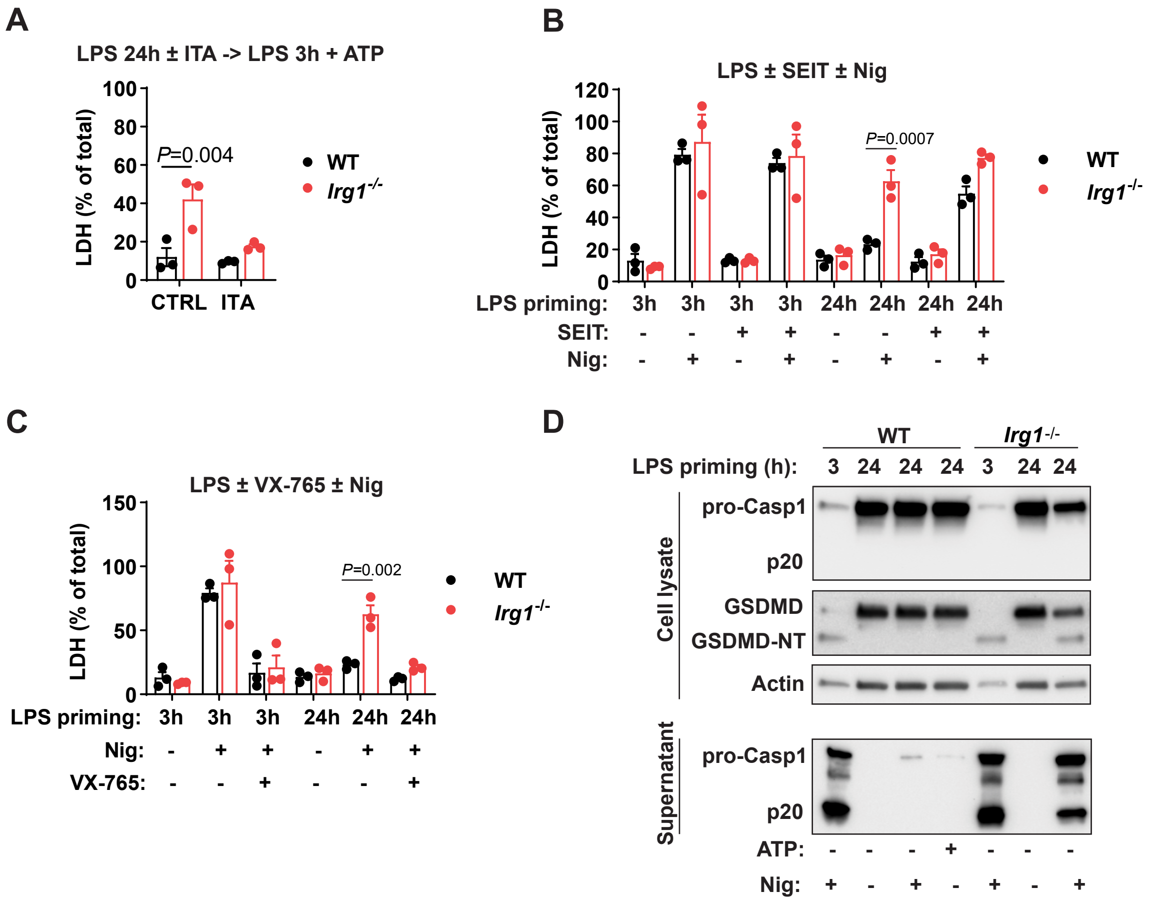

Figure S5. Itaconate regulates pyroptosis after sustained LPS stimulation. Related to Figure 5. (**A**) Lactate dehydrohenase (LDH) activity in supernatants of BMDMs stimulated as in Figure 3A, ATP was added for 2 h, n = 3 experiemtns. (**B**) LDH activity in supernatants of BMDMs stimulated with LPS for 3 or 24 h followed by nigericin (Nig) for 2 h (as in Figure 5A). SEIT (500 μ M) was added at 1 h of LPS stimulation, n = 3 experiments. (**C**) LDH detection in the supernatants of BMDMs stimulated as in Figure 5A. Caspase-1 inhibitor VX-765 (50 μM) was added 30 min before stimulation with Nig, n = 3 experiments. (**D**) Western blot detection of caspase-1 (Casp1) and GSDMD forms in whole cell lysates and supernatants sof BMDMs timulated as in Figure 5A. Representative of n = 3 experiments. Actin was used as loading control. *P* values was calculated using 2-way ANOVA with Sidak's test. Data represent mean + SEM.

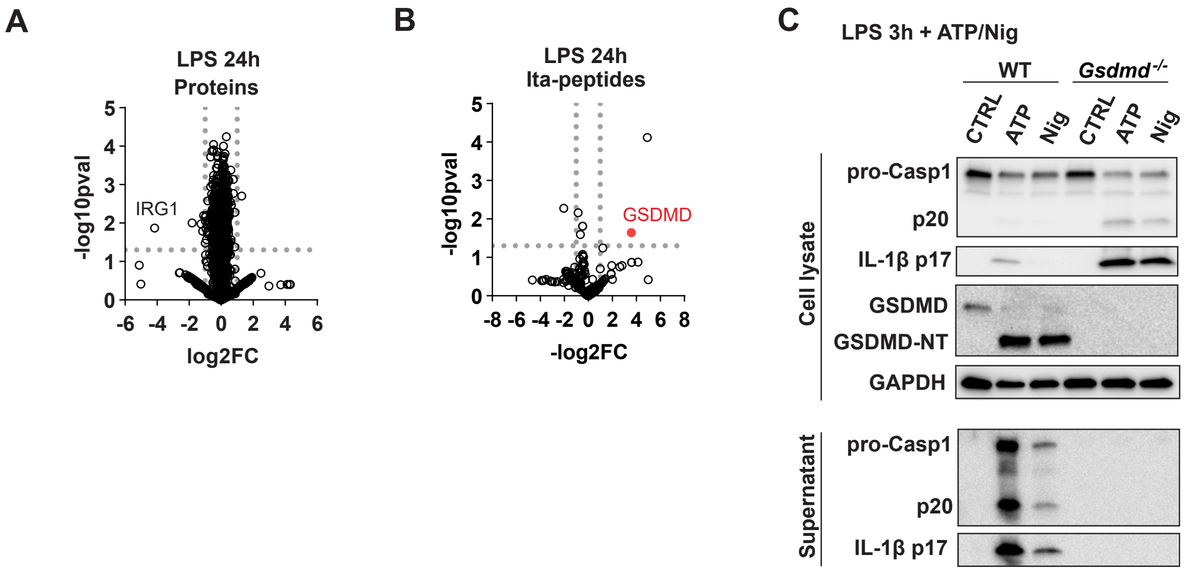

Figure S6. GSDMD is a gatekeeper of late pyroptosis and is itaconated in activated macrophages. Related to Figure 6. (**A**, **B**) Changes in global protein (A) and itaconated peptide (Ita-peptide; B) content in BMDMs treated with LPS for 24 h detected by mass spectrometry; data are mean from n = 6, 3 cultures from 2 experiments; *P* values were determined using two-tailed, unpaired, Student's t-test. (**C**) Detection of proteins in cell lysate and supernatant of BMDMs stimulated with LPS for 3 h and with ATP or nigericin (Nig) for 45 min. GAPDH was used as loading control. Representative of n = 3 experiments.

Table S2. Itaconated peptides detected by mass spectrometry. Related to Figure 6. BMDMs were stimulated with LPS as in Figure 1H, except no ATP was added, n = 6, 3 cultures from 2 experiments.

Gene Name	MPA	FC	pval	Annotated Sequence	Modification
Ifi30	Q9ESY9	0.00861	0.000324	[R].VSLYYESLCGACR.[Y]	C9(Cys-Ita); C12(Carbamidomethyl)
Gsdmdc1	Q9D8T2	0.026489	0.001914	[K].DILEPSAPEPEPECFGSFK.[V]	C14(Cys-Ita)
Gapdh	P16858	0.03978	1.28E-07	[R].AAICSGK.[V]	C4(Cys-Ita)
Ldha	P06151	0.043772	1.96E-05	[K].DYCVTANSK.[L]	C3(Cys-Ita)
Eef2	P58252	0.044818	0.051727	[K].STLTDSLVCK.[A]	C9(Cys-Ita)
Ctsb	P10605	0.054648	6.7E-05	[R].DQGSCGSCWAFGAVEAISDR.[T]	C5(Carbamidomethyl); C8(Cys-Ita)
Cfl1	P18760	0.073819	2.46E-05	[K].HELQANCYEEVK.[D]	C7(Cys-Ita)
Glrx	Q9QUH0	0.163789	0.006052	[K].VVVFIKPTCPYCR.[K]	C9(Cys-Ita); C12(Carbamidomethyl)
Ctsb	P10605	0.397523	0.026327	[R].DQGSCGSCWAFGAVEAISDR.[T]	C5(Cys-Ita); C8(Carbamidomethyl)
Lyz2	P08905	0.625137	0.041528	[R].AVNACGINCSALLQDDITAAIQCAK.[R]	C5(Cys-Ita); C23(Carbamidomethyl)

MPA, master protein accession; FC, fold change; pval, *P* value.

Figure 1C

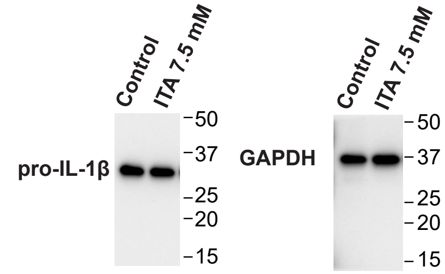

Figure 1G

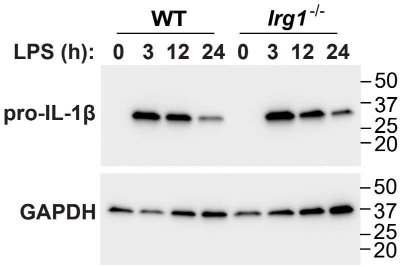

Figure 1J

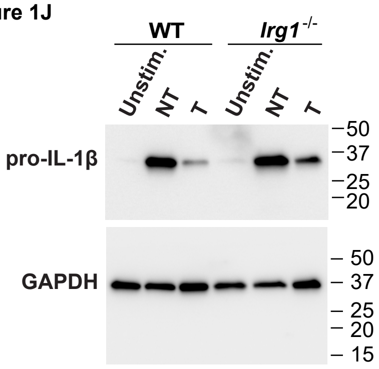

Figure 2B

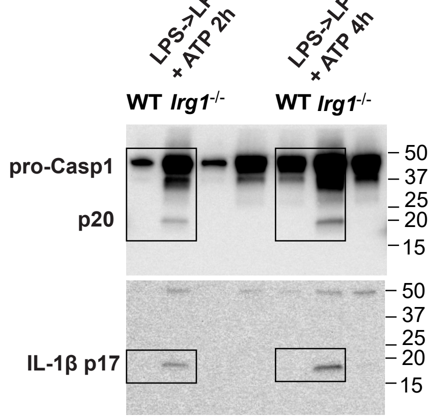

Figure 2D

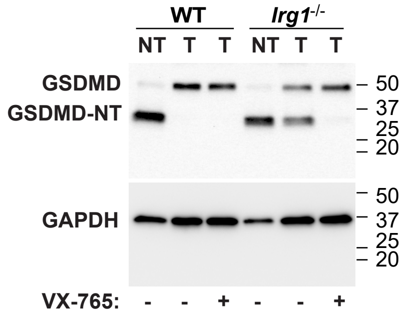

Figure 3C

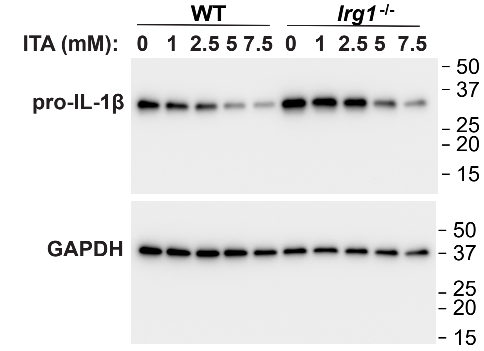

Figure 3F

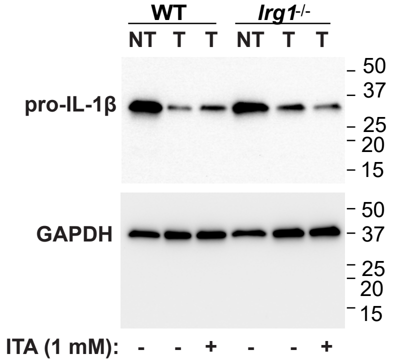

Figure 3G

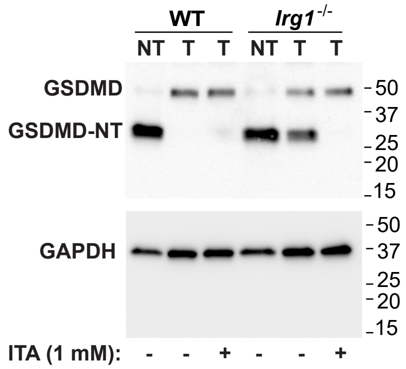

Figure 4B

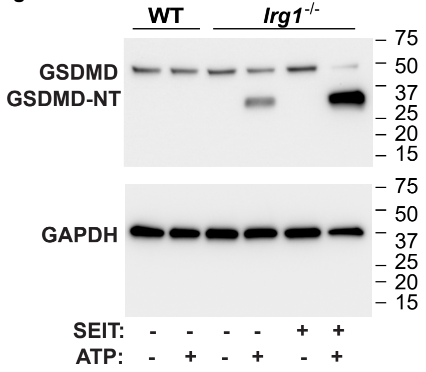

Figure 4E

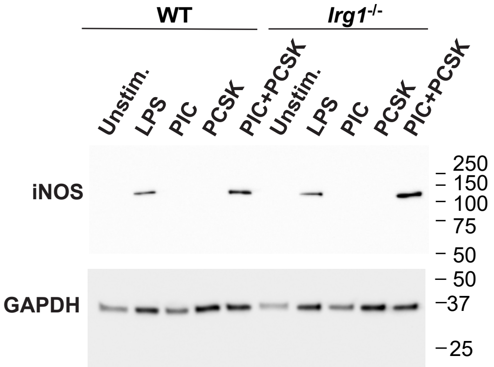

Figure 4H

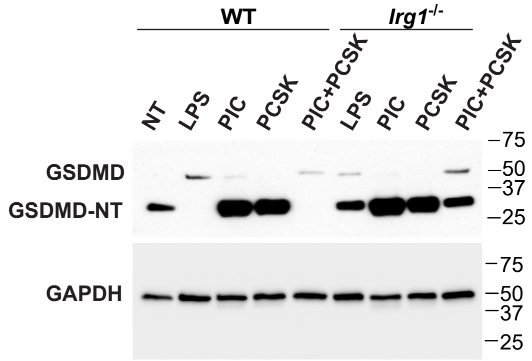

Data S1. Full western blot images. Related to Figures 1-5, S2-S6.

Figure 4J

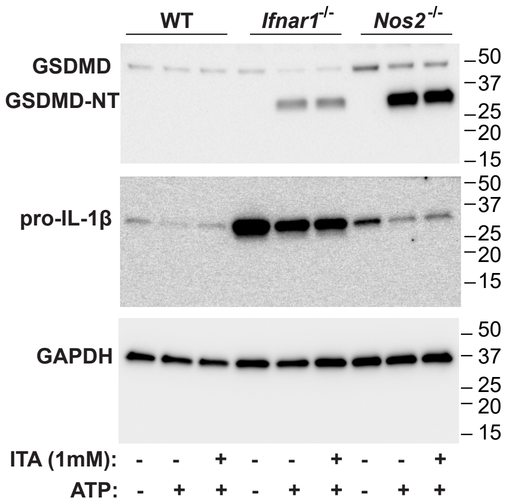

Figure 5E

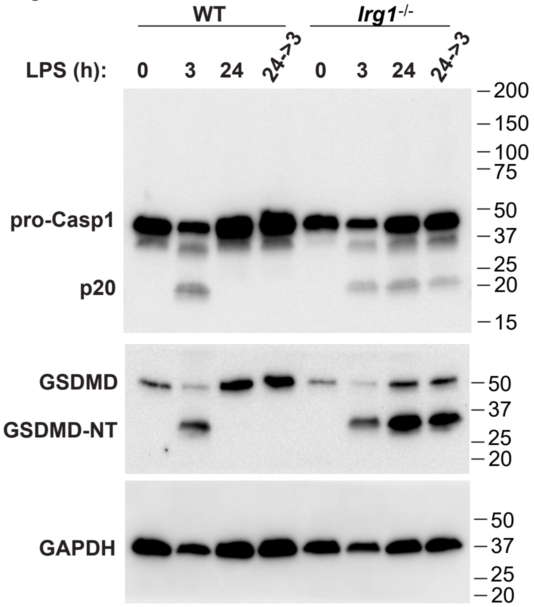

Figue S2E

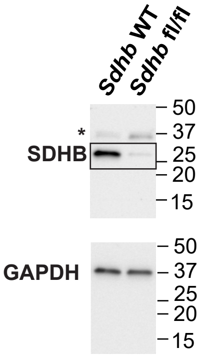

Figure S3D

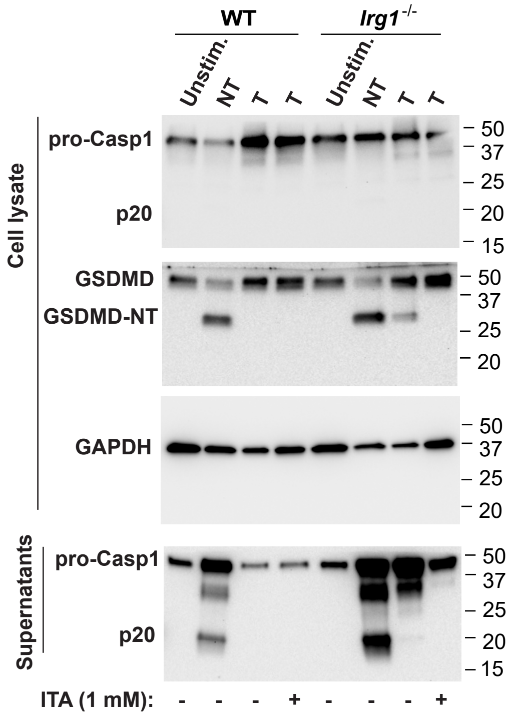

Figure S3E

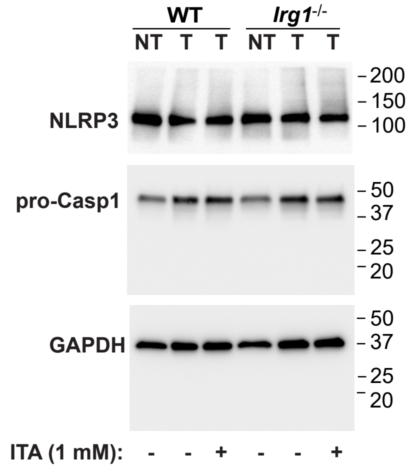

Figure S3F

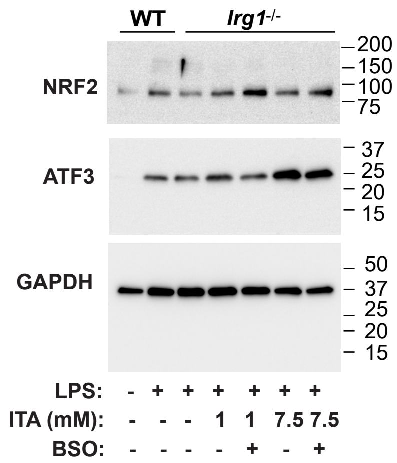

Figure S4A

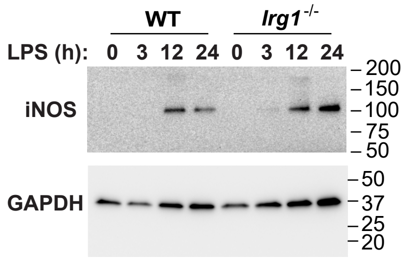

Data S1. Full western blot images. Related to Figures 1-5, S2-S6.

Figure S4C

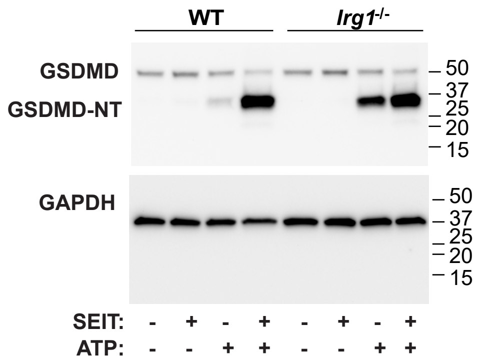

Figure S4H

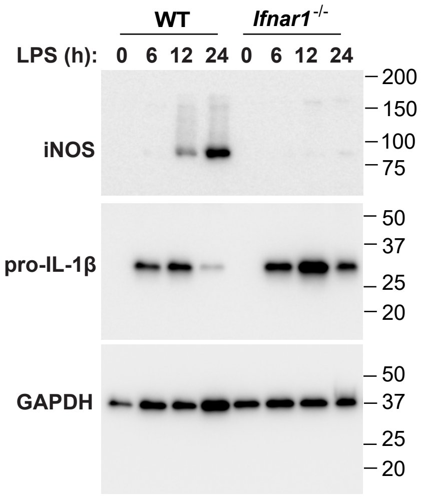

Figure S5D

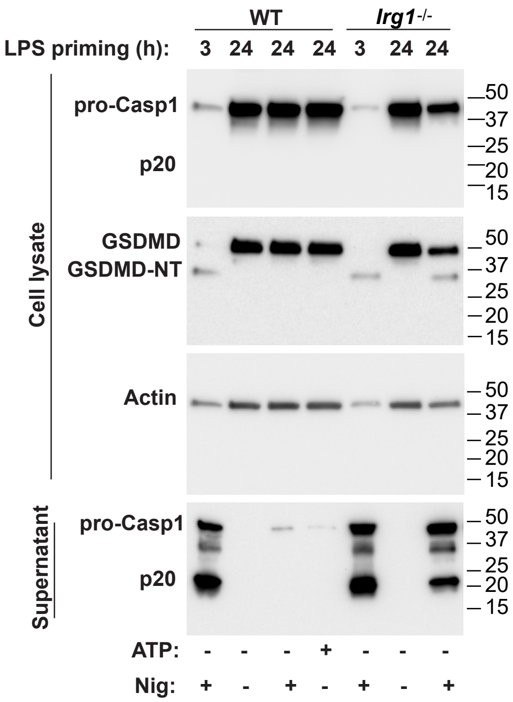

Figure S6C

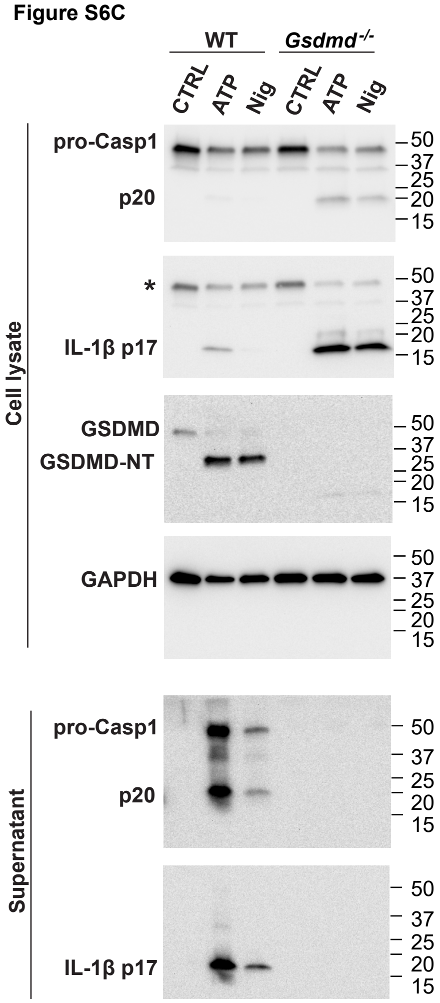

* Band reminiscent from previous developing.

Data S1. Full western blot images. Related to Figures 1-5, S2-S6.

T cell panel

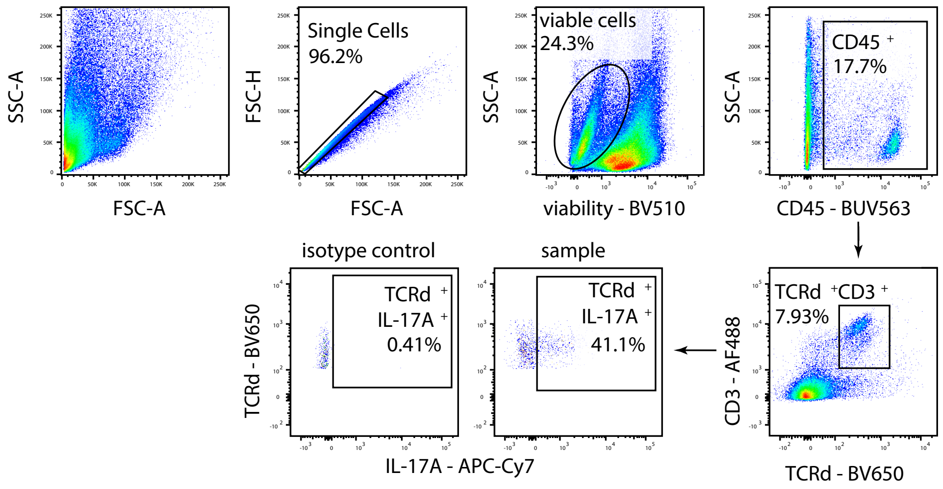

Myeloid panel

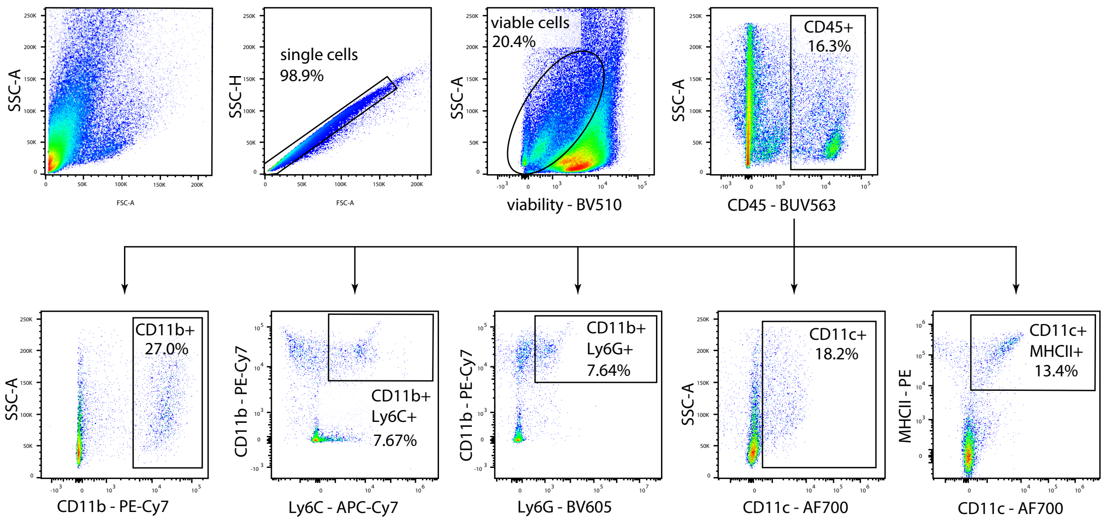

Data S2. Gating strategy. Related to STAR Methods.
